# Spiking Neural Networks with Continual Learning for Steering Angle Regression: A Sustainable AI Perspective

**DOI:** 10.3390/s26092656

**Published:** 2026-04-24

**Authors:** Fernando S. Martínez, Sergio Costa, Raúl Parada

**Affiliations:** 1 e-Health Center, Universitat Oberta de Catalunya (UOC), 08018 Barcelona, Spain; fsevillama@uoc.edu (F.S.M.); scostap@uoc.edu (S.C.); 2 Centre Tecnològic de Telecomunicacions de Catalunya (CTTC/CERCA), Av. Carl Friedrich Gauss 7, Castelldefels, 08860 Barcelona, Spain

**Keywords:** spiking neural networks, continual learning, autonomous driving, neuromorphic computing, V2X communication, edge AI, sustainable AI, catastrophic forgetting, energy efficiency, federated learning

## Abstract

This work explores the application of Spiking Neural Networks (SNNs) and Continual Learning (CL) methodologies to the problem of steering angle regression, using autonomous driving simulation as the experimental context, with a focus on energy efficiency and alignment with sustainable computing objectives. The primary goal was to design and implement CL techniques in SNNs to assess the model’s ability to maintain accuracy in explored environments while reducing CO_2_ emissions through the optimized use of a subset of the data. This study emerges in response to the increasing energy demand of deep learning models, which poses a challenge to sustainability. SNNs, inspired by the efficiency of biological neural systems, offer significant advantages in terms of computational and energy consumption, making them a promising alternative. CL techniques, such as Elastic Weight Consolidation and replay memory, are integrated to mitigate catastrophic forgetting in sequential learning tasks. The methodology includes adapting the PilotNet architecture for SNNs, preprocessing datasets generated in the Udacity driving simulator, and evaluating models in incremental learning scenarios. The experiments compare the performance of SNNs with CL against baseline models without CL, using mean squared error (MSE), computational efficiency, and equivalent CO_2_ emissions as evaluation metrics. The results demonstrate that replay memory enables the retention of prior knowledge with a limited increase in energy consumption. This work concludes that SNNs with CL are a viable alternative for sustainable AI applications. Future research directions include a focus primarily on hardware-specific implementations and real-world testing.

## 1. Introduction

Deep learning models have experienced exponential growth in almost all sectors of society, from autonomous driving (AD) to healthcare, industry, and energy. However, this expansion poses a significant energy challenge due to the high power consumption associated with the training and deployment of these models, particularly in the case of deep neural networks [1].

This underscores the need to explore more energy-efficient alternatives that reduce the carbon footprint of Artificial Intelligence (AI), while aligning technological advances with the Sustainable Development Goals (SDGs).

### 1.1. Sustainable AI Framework: Definition and Quantification

In this work, Sustainable AI is operationalized through three measurable dimensions, each grounded in empirical evidence derived from our prior comparative study [2].

Sustainability is approached not as a purely conceptual notion, but as an actionable framework that involves evaluating computational capability, energy demands, and adaptability within the constraints of incremental learning. Firstly, computational efficiency is characterized as the optimization of operations necessary for each unit of model performance. This dimension is assessed through three complementary metrics. FLOPs-per-prediction reveal that SNNs achieve a 2–3× reduction compared to CNNs when spike sparsity is properly considered. Importantly, this reduction is not confounded by architectural scaling, since both SNN and CNN variants maintain identical parameter counts (252 K parameters for PilotNet), ensuring a fair comparison in terms of representational capacity. However, the encoding strategy plays a decisive role in the efficiency of the data representation; while Rate encoding with S=25 increases the input data volume by 25×, Delta encoding preserves sparsity and therefore maintains a substantially lower effective computational load.

Secondly, energy consumption is measured directly during both training and inference using CodeCarbon [3], enabling hardware-aware and resource-complete accounting. The baseline measurements reported in [2] show that CNN consumes 0.2 mWh per validation epoch, while SNN with Delta encoding reduces this value to 0.1 mWh per validation epoch, representing a 50% reduction. In contrast, the SNN with Rate encoding requires 2.1 mWh per validation epoch, corresponding to an increase of 10× relative to the CNN baseline. These measurements incorporate the total utilization of computational resources, which includes the CPU, GPU, and RAM. Energy consumption (Wh) is the primary sustainability metric reported throughout this work. Where CO_2_ equivalents are cited, they are derived from the carbon intensity of the local electrical grid at the time and location of the experiments (Germany, ≈400 gCO_2_/kWh, sourced via the Electricity Maps API as reported by CodeCarbon [4]), and should be interpreted as locally contextualized estimates rather than universal values [4].

Thirdly, sustainability extends beyond static efficiency to adaptability without retraining. By enabling incremental knowledge through Continual Learning (CL), the model mitigates the substantial energy costs generally associated with repeated full retraining cycles. For *n* sequential tasks, a naive strategy requires *n* complete retraining procedures, resulting in a total energy cost of $n\times E_{\mathrm{full}}$. In contrast, a rehearsal-based strategy performs *n* incremental updates with an energy cost approximated by $\left(n\times E_{\mathrm{task}}\right)\times \left(1+r\right)$, where r<0.25 represents the rehearsal buffer ratio. Under realistic assumptions, this results in a projected 65% energy reduction for Task 2, which is scalable to more than 80% savings by Task 5 and subsequent tasks. Together, these three dimensions provide a structured and empirically grounded framework for evaluating Sustainable AI in sequential learning settings.

#### 1.1.1. Quantitative Framework

The sustainability assessment in this work is based on three complementary metrics. Predictive accuracy is measured through the Mean Squared Error (MSE) on the validation sets of both tracks. The computational cost is quantified through training time and equivalent CO_2_ emissions, measured using the CodeCarbon library [3]. Finally, the degree of catastrophic forgetting is evaluated through the Forgetting Ratio (FR). Together, these metrics allow for a joint assessment of performance and eco-efficiency across all evaluated CL strategies. A composite energy-accuracy metric, the Energy-to-Error Ratio, was used in our prior comparative study [2] and is referenced where relevant for contextualization with those results.

#### 1.1.2. Synergistic Effects

A conclusion of this work is that the integration of SNNs with CL produces a sustainability multiplicative benefit, rather than simply additive. The SNN architecture provides a baseline efficiency gain of approximately 2× in per-inference energy consumption compared to equivalent CNNs [2], while the rehearsal-based CL strategy reduces the total adaptation energy by a factor of 2.85 compared to naive retraining, as demonstrated in the experiments reported here. The combined effect amounts to approximately 5.7× total energy reduction for two-task scenarios. Moreover, this advantage compounds as the number of tasks grows; projections for ten sequential tasks suggest an energy reduction on the order of 18× compared to naive CNN retraining, underscoring the practical relevance of the proposed approach for real-world AD deployments.

This framework positions sustainability not as a single optimization objective but as an emergent property of architectural choice (SNNs), encoding strategy (Delta), and learning methodology (Rehearsal-based CL). The integration of these components, validated through rigorous energy measurement and statistical analysis, constitutes the primary contribution of this work to sustainable AI in autonomous driving applications.

This approach aims to contribute to current research in neuromorphic models, providing valuable insights for both academic development and practical applications in intelligent and sustainable AD systems.

The remainder of this paper is organized as follows. Section 2 reviews the current state of the art, focusing on recent advancements in SNNs and CL, particularly in regression tasks. Section 3 presents the theoretical foundations of SNNs and CL, highlighting their relevance in energy-efficient and adaptive autonomous systems. It also details the proposed methodology, including the data description and preprocessing strategies, and describes the experimental setup, outlining the simulation environments, CL scenarios, evaluation metrics, and training configurations used to assess model performance. The results of these experiments, along with a comprehensive analysis of predictive accuracy, energy efficiency, and resilience to catastrophic forgetting of SNN models, are presented in Section 4. Finally, Section 5 summarizes the key findings and discusses their implications for sustainable AI development. In addition, future research directions are discussed, focusing on hardware-specific implementations and real-world validations to further advance the applicability of SNNs and CL in AD systems.

## 2. State of the Art

This section reviews recent studies on CL applied to SNNs in regression tasks, analyzing their approaches and results.

### 2.1. Continual Learning Applied to SNNs

The application of SNNs has seen considerable growth in recent years. However, the distribution of research efforts across different neural network tasks has not been uniform. As a result, the number of publications that focus on the application of SNNs to classification tasks far exceeds those that address other use cases [5].

This bias is evident in review studies on SNNs, such as that of [6], as well as in research on CL applied to SNNs, as exemplified by [7]. Fewer studies have been conducted on continuous value prediction (or regression), such as [5]. The most common applications of SNNs in regression tasks involve time series prediction, particularly in financial markets and environmental modeling, such as meteorological and climate forecasting, as demonstrated in that same study.

In this context, the work of [8] is particularly relevant as it introduces a novel framework to use SNNs in regression tasks. Specifically, the authors propose using the membrane potential of the output layer to approximate continuous values, applying and validating this approach in industrial material mechanics to predict material behavior.

Despite the limited research on the application of SNNs to regression tasks, an even larger gap is observed in the application of CL methods to regression tasks using SNNs, where no prior studies have been found. Table 1 summarizes recent and related works that share similarities with the present study.

A review of the most relevant literature in the field to this study highlights significant knowledge gaps at the intersection of SNNs applied to regression problems and CL. These gaps underscore the significance of the contribution of this study:

- The study aims to implement the most recent SNN training strategies, particularly backpropagation through time using surrogate gradients.
- To address the proposed regression problem, the membrane potential will be utilized, a methodology that represents the state of the art in SNNs applied to regression.
- The implementation of catastrophic forgetting mitigation strategies in SNNs within a CL setting in regression problems is an innovative approach for which no prior research has been found. However, this direction is crucial for reducing the energy consumption of AI systems.
- Finally, applying the above strategies in an AD framework, even in a simulated environment, introduces a novel evaluation setting for SNNs. This work also lays the foundation for real-world implementations and future applications.

### 2.2. SNNs in Classification Versus Regression: Distinct Challenges

While SNNs have demonstrated remarkable success in vision-based classification tasks, including object detection [13], semantic segmentation [14], and edge detection [15], their application to regression problems presents fundamentally different challenges that explain the limited research in this area.

Why Classification Succeeds:

Classification tasks exhibit a natural congruence with the discrete, spike-based characteristics of SNNs for multiple reasons. The outcome of a classifier necessitates the identification of the neuron exhibiting the highest level of activity, which correlates directly with spike counts or the timing of the first spike, thereby eliminating the requirement for precise continuous-valued outputs. Moreover, the classification of visual stimuli is enhanced by the event-driven processing of spatial attributes such as edges and textures, which can be succinctly encoded as binary spike patterns. Finally, since classification decisions can aggregate information over a temporal window, occasional spike irregularities have a limited impact on the final prediction, making the approach inherently robust to the stochastic nature of spiking activity.

Regression Challenges:

Regression tasks in AD, such as predicting continuous steering angles, present a fundamentally different set of challenges [8]. Unlike classification, where the output corresponds to the most active neuron, regression requires encoding a continuous range of values, for example, from −25° to +25°, from membrane potentials or spike rates. This requires precise calibration and is sensitive to encoding noise in a way that classification is not. Training is also more demanding. Although surrogate gradients provide a workable approximation for classification, where discrete decisions tolerate some gradient imprecision, regression requires higher gradient accuracy to minimize a continuous MSE loss. Related to this, the typical output layer design for regression relies on a single neuron whose membrane potential encodes the predicted value, which is more sensitive to parameter variations and offers less redundancy than the multi-neuron output used in classification. Two further challenges arise in the context of AD specifically. First, steering predictions must be smooth and temporally consistent across frames, since spike irregularities that are inconsequential in classification can translate into undesirable jitter in the control signal. Second, the MSE loss landscape is smoother than cross-entropy but lacks the discrete class boundaries that act as anchors during optimization, making convergence more difficult when gradients are only approximated.

Implications for This Work:

The limited implementation of SNN in regression applications in AD is mainly due to these technical challenges rather than to the fundamental limitations of SNNs. The present work addresses these obstacles through several methodological choices. Firstly, rate encoding with a sufficient number of timesteps (S=25) is employed to ensure a stable and reliable continuous value representation. Secondly, regression outputs are obtained directly from the membrane potential of the output LIF neuron, enabling continuous-valued prediction without requiring spike counting post-processing. Thirdly, surrogate gradient BPTT is applied, accompanied by systematic hyperparameter tuning (see Section 3.6.1) to ensure stable optimization. Finally, validation is performed through two distinct tracks characterized by varying degrees of complexity to evaluate the capacity for generalization under diverse driving conditions. Importantly, the successful integration of CL within an SNN-based regression framework in this study demonstrates that these technical challenges can be effectively mitigated. The results establish a foundation for advanced studies in the context of neuromorphic autonomous systems. Future research should investigate whether the intrinsic temporal processing capabilities of SNNs, currently underexploited in the adopted rate-coding scheme, could yield additional benefits in regression-oriented time-series prediction tasks, particularly in scenarios where anticipating steering angles several frames ahead would provide operational advantages.

## 3. Materials and Methods

This section presents the theoretical foundations of this work. First, we introduce SNNs and highlight the main features that distinguish them from second-generation ANNs. Then, we discuss the concept of CL and describe the main methodologies used for its implementation in neural networks. Finally, we describe the dataset used in this study and design the experiments to evaluate performance.

### 3.1. Spiking Neural Networks

SNNs represent a step forward in the biological plausibility of ANNs and are considered the third generation of ANNs [16]. They differ primarily in their use of spiking neurons, which incorporate a state variable (membrane potential) and a digital signaling mechanism (action potentials) and unlike conventional ANNs, which operate with continuous values, SNNs use binary activations (spikes) to encode information in terms of timing and frequency, more closely resembling how the brain processes [16,17].

From a theoretical perspective, ref. [17] highlight that the advantages of SNNs over traditional ANNs stem from three key neurobiological principles: (i) spiking activity, spiking activity, which when modeled require only summations and weight identity multiplications in the presence of a spike and significantly reduces the computational overhead while maintaining meaningful information time encoded; (ii) sparsity as neurons remain inactive most of the time, allowing for more efficient memory usage and processing compared to ANNs; and (iii) static suppression, as SNNs process primarily dynamic information, reducing redundancy and improving efficiency.

Despite these advantages, the implementation of these neurobiological principles poses several challenges that must be addressed to realize the full potential of SNNs [17,18]: approximation error during encoding; non-differentiable activation function in spiking neurons that prevents direct gradient computation during standard backpropagation-based optimization methods; and asynchronous read/write operations for neuron states which increases overall system complexity.

Current research efforts are focused on overcoming these limitations to develop more robust and efficient SNNs for a wider range of applications.

Depending on their focus on biological plausibility, SNN research can be categorized into two main approaches: (1) Studies that aim to closely mimic neurobiological processes to better understand brain function. (2) Studies that prioritize the development of efficient network architectures and training methods, even at the expense of biological fidelity.

While both approaches contribute significantly to the field, their goals and methodologies are quite different. Since this work primarily falls into the second category, the following sections will focus on studies relevant to this area.

#### 3.1.1. Mathematical Model of Spiking Neurons

The mathematical models that define spiking neurons are fundamental to the functionality of SNNs. Among the diverse neuron models, the Leaky Integrate-and-Fire (LIF) Model is the most widely used model in SNNs due to its balance between computational simplicity and ability to capture temporal neuronal dynamics [17,19].

The operation of an LIF neuron is based on four key principles [19]:

- **Integration:** The neuron accumulates input signals in its membrane potential. Each incoming spike contributes to a change in potential.
- **ILeakage:** Unlike ideal neurons, where the membrane potential remains constant in the absence of stimuli, LIF neurons experience a gradual decay over time, modeled by a time constant (τ).
- **Firing:** When the membrane potential exceeds a threshold ($V_{th}$), the neuron generates an output spike, which sends a signal to other neurons. After firing, the membrane potential returns to a predefined value.
- **Refractory Period:** After firing, the neuron may enter a short refractory period during which it cannot fire again, mimicking the behavior of biological neurons.

#### 3.1.2. Signal Encoding in Spiking Neural Networks

Signal encoding is a fundamental aspect of SNNs because it determines how information is represented as spike trains. Spiking neuron models, such as the LIF model discussed earlier, operate on discrete-time signals, unlike ANNs, which use analog values. Consequently, signal encoding plays a critical role in the conversion of analog input data into spike trains and in the interpretation of network outputs, as explained in [17]. The main signal encoding methods include the following, as detailed in the same study: (i) rate encoding, the most widely used coding method in SNNs, where information is represented by spike frequency over time; (ii) latency encoding, which encodes information on the timing of the first spike; (iii) delta modulation encoding, which generates spikes only when the input intensity changes; and (iv) other encoding methods including direct encoding, where data is not pre-encoded, temporal pattern encoding and population encoding. The latter methods introduce additional complexity that can make implementation and interpretation more difficult, and the increased computational cost limits their use to specific contexts where the benefits outweigh these challenges. Although rate encoding can be slower and yield higher power consumption, it provides high error tolerance and facilitates training using backpropagation algorithms, as a higher spike count provides a better approximation of the gradient.

#### 3.1.3. Training SNNs

Training SNNs presents unique challenges due to the non-differentiable nature of the activation functions used by spiking neurons, as discussed above. The discrete behavior of information transfer within the network complicates the direct application of gradient-based learning methods such as backpropagation, which is a standard approach for training deep networks [17]. To overcome these limitations, several alternative methods have been proposed, which can be broadly categorized into three main approaches:

- **ANN-to-SNN Conversion:** An ANN with the same architecture as the target SNN is defined and trained using standard backpropagation techniques. Once trained, it is transformed into an SNN by replacing artificial neurons with spiking neurons [17,20]. This approach is also referred to in some literature as “offline conversion” or “weight transfer” methods.
- **Spike Timing Dependent Plasticity (STDP):** STDP is a biologically plausible local learning rule that adjusts synaptic weights based on the timing of spikes between pre- and postsynaptic neurons [21]. This technique captures the causal relationship inherent in spike timing, making it particularly effective in unsupervised learning tasks [22]. Despite its potential, STDP faces limitations, particularly in scaling to deep SNN architectures and applying STDP to supervised learning tasks.
- **Backpropagation-Based Training:** To take advantage of the well-established training and optimization algorithms developed for ANNs, it is necessary to address the non-differentiability problem. The Surrogate Gradient (SG) technique replaces the activation function during backpropagation with an approximation that is both differentiable and sufficiently close to the original function. This approximation allows for gradient computation and backpropagation through the network layers [17]. Despite their effectiveness, SG methods may face limitations when deployed on large-scale neuromorphic hardware due to the high memory and energy requirements of these systems [23]. A widely used method is Backpropagation Through Time (BPTT), similar to its application in Recurrent Neural Networks (RNNs), unfolds the network over time, treating each time step as a layer in a feedforward network. This approach allows the error to propagate backward through time, allowing the characterization of individual contributions at each time step and enabling the network to learn from temporal dependencies within the data. Despite its advantages, one of the main challenges is the high memory consumption required to store membrane potentials for all *S* and the increased computational requirements, especially for long time sequences, that can lead to longer training times when scaling network architectures [24]. These factors highlight the need for careful resource management and optimization strategies when implementing BPTT in large-scale SNNs.

### 3.2. Continual Learning

CL (or lifelong learning) in the context of neural networks refers to the ability of these networks to continuously acquire and integrate new knowledge (plasticity) without forgetting previously learned information (stability). This concept is inspired by the ability of humans and other organisms to adapt constantly to new environments and acquire new skills without losing previously acquired abilities [25].

In machine learning, it is typically assumed that training data are independently and identically distributed (i.i.d). This assumption implies that each data point is drawn from the same probability distribution and that the data points are independent of one another. However, in real-world scenarios, this assumption is often not upheld, as data distributions may shift over time, a phenomenon known as concept drift. This poses a significant challenge for models, as training under i.i.d conditions does not prepare them to handle distributional changes, leading to poor generalization and degraded model performance [25,26]. Therefore, finding an optimal balance between plasticity and stability is crucial, a challenge commonly referred to as the stability-plasticity dilemma. In practice, the effectiveness of CL methods is often evaluated using standardized benchmarks and metrics such as forgetting, transfer, and efficiency, which provide a unified framework for comparing approaches [27].

Retraining a model from scratch each time it encounters new data is considered an inefficient and impractical solution, particularly in real-world applications where computational resources are limited. However, in a sequential training setting, learning new tasks or concepts may lead to the forgetting of previously acquired knowledge, a phenomenon known as catastrophic forgetting. This occurs because the model parameters are optimized for the current data distribution, which may conflict with parameters previously optimized for earlier data distributions [25].

#### 3.2.1. Continual Learning Scenarios

To address the problem of catastrophic forgetting in CL scenarios, it is essential to define the training and inference settings for the given task. In this context, a widely adopted classification, originally proposed by [28,29], and later reinforced by [30], distinguishes between three main CL scenarios:

- **Class-Incremental Learning (Class IL):** New classes are introduced over time, requiring the model to learn them incrementally. This scenario is common in real-world applications, where the set of classes to be classified expands as new data become available.
- **Domain-Incremental Learning (Domain IL):** The input distribution changes while the output labels remain the same. This scenario is typical in applications such as speech recognition or object detection, where recordings or images may originate from various outdoor environments.
- **Task-Incremental Learning (Task IL):** Entirely new tasks are introduced sequentially. This setting is common in multipurpose systems that need to expand their capabilities over time.

Although some authors have proposed additional categories to encompass a wider range of scenarios, such as task-agnostic learning [31] or open-world learning [32], the classification outlined above covers the vast majority of practical CL applications [30].

#### 3.2.2. Continual Learning Methods

There are three main families of methods for addressing CL, as [30] describe in their work:

- **Regularization-Based Methods:** Aimed to preserve previously learned knowledge by imposing constraints on model updates, such as parameters, hyperparameters, and activations. This is achieved by introducing additional regularization terms into the loss function, penalizing drastic changes in parameters that are crucial for previously learned tasks. By minimizing weight and activation drift, these methods help mitigate catastrophic forgetting.
- **Parameter Isolation-Based Methods:** This family of methods seeks to minimize task interference by assigning parameter subsets to specific tasks. As a result, modifications to parameters associated with one task do not negatively impact the performance of previously learned tasks. Within this category, dynamic architecture methods expand the neural network to accommodate new tasks by adding neurons, layers, or modules to the structure. In contrast, fixed architecture approaches maintain a constant model size while partitioning parameters into global and task-specific spaces.
- **Memory-Based Methods:** These methods store information about previous tasks in an external memory for future reuse. Some approaches retain a subset of the original data for later replay (Rehearsal), which in certain cases may raise data privacy concerns. Alternatively, privacy-preserving methods store memory vectors, feature representations, or gradient spaces that encapsulate task information without directly storing raw data. Another variant, generative replay methods, leverage generative networks to synthesize past data for replay without the need to retain the original datasets.

The selection of the most suitable method depends on factors such as the nature of the tasks, available resources, and specific application requirements. According to [30], combining methods from different categories may be an effective strategy to achieve more robust and generalizable CL.

### 3.3. Dataset

For this study, the Udacity simulator [33], an educational tool designed to train autonomous vehicles through CNN, is used to generate the dataset. The data is acquired by manually driving a simulated vehicle in the simulator.

#### 3.3.1. Dataset Description

The simulator provides two tracks with distinctive characteristics:

**Track 1:**

- **Terrain type:** Paved road without lane markings, featuring gentle curves, moderate straights, and a small stone bridge.
- **Elevation changes:** Contains slight ascents and descents, simulating a suburban road environment.
- **Weather and lighting conditions:** Simulates a sunny day with consistent lighting conditions.

**Track 2:**

- **Terrain type:** Paved road with lane markings, sharper curves, and narrower sections, offering a more challenging environment.
- **Elevation changes:** Includes steeper inclines and declines, simulating a secondary road through a jungle setting.
- **Weather and lighting conditions:** Simulates mid-morning or late-afternoon conditions, with sunlight variations affecting visibility and creating long shadows.

**Figure 1:**
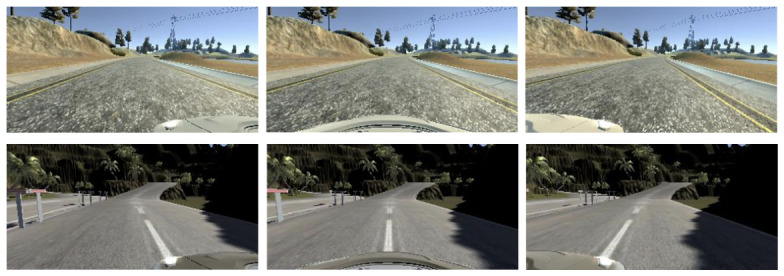
Sample images of Track 1 (**above**) and Track 2 (**below**).

The simulated vehicle is equipped with three front-facing cameras: one central and two lateral (left and right). These cameras capture images during the simulation, generating a visual dataset that reflects different perspectives of the road, as illustrated in Figure 1.

Additionally, the simulator generates a log file containing the following information for each recorded image:

- **Image paths:** Specifies the file paths of images captured by the central, left, and right cameras (stored in the first three columns, respectively).
- **Steering angle:** A numerical value indicating the steering angle of the vehicle at the moment the image is captured. Positive values represent right turns, while negative values indicate left turns, normalized within the range of −1 to +1 (corresponding to turns between −25 and +25 degrees).
- **Throttle:** Measures acceleration in mi/h^2^.
- **Brake:** Reflects deceleration applied through braking, also in mi/h^2^.
- **Speed:** Records the velocity of the vehicle in mi/h.

The initial dataset consists of 15,748 samples for Track1 and 5198 samples for Track 2, collected by manual driving using keyboard inputs. These data include a total of 24,108 and 15,594 images, respectively, stored in RGB format, with a resolution of 320 × 160 pixels and a frame rate of 10 images per second.

**Figure 2:**
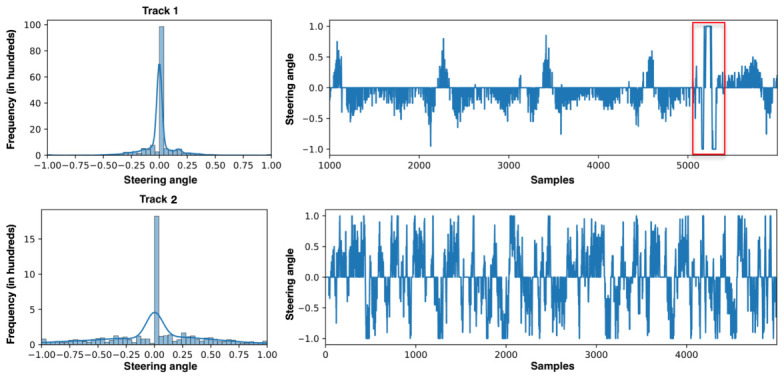
(**Left**): Histogram of steering angle values. (**Right**): Steering angle distribution during the simulation for the first 5000 samples. Track 1 and Track 2 are shown in the upper and lower positions, respectively. The red box highlights a direction change.

Figure 2 presents steering angle distribution and variation over 5000 consecutive samples for comparison.

In Track 1, the temporal sequence exhibits a clear repetitive pattern approximately every 1000 samples, reflecting the laps completed on the track. A total of seven laps were recorded in the counterclockwise direction and five laps in the clockwise direction.

The track analysis also reveals an imbalance in the number of curves depending on the direction: there are more left-hand turns than right-hand turns. This imbalance is mitigated by navigating in opposite directions, which helps partially balance the data distribution.

Track 2 presents a more homogeneous distribution of curves, with shorter and sharper turns compared to Track 1. This track is also longer, and the simulation includes two complete laps. This configuration results in a more balanced variability in the steering angles.

With regard to the data distribution of both tracks, the histograms show a clear predominance of angles close to zero, which is to be expected as the vehicle travels in a straight line for a significant portion of the simulations, although it is less pronounced in track 2.

This predominance poses a challenge for model training, as it may introduce a bias toward neutral values and could lead to a loss of sensitivity in the curves, as the model is exposed to these cases less frequently during training. This issue will be addressed in the following section.

#### 3.3.2. Dataset Preprocessing

**Figure 3:**
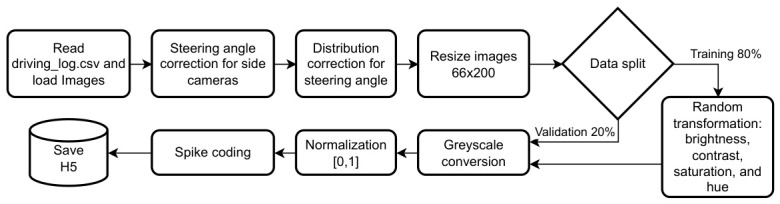
Flowchart illustrating the preprocessing and encoding of data.

To align with the objectives of this study and minimize computational time and cost during training and testing, data preprocessing, augmentation, and spike encoding will not be performed on the fly. Instead, these steps will be carried out in advance, and the resulting data will be stored in h5 files for use in subsequent stages. The full pipeline is outlined in the flowchart shown in Figure 3.

##### Correction of Steering Angle for Lateral Cameras

The simulator generates a triplet of images (central camera, left lateral camera, and right lateral camera) for each captured sample. To leverage this different perspectives, each camera will be treated as an independent sample, as this approach increases both the quantity and diversity of samples, improving the representation of curves and enriching the dataset for model training.

**Figure 4:**
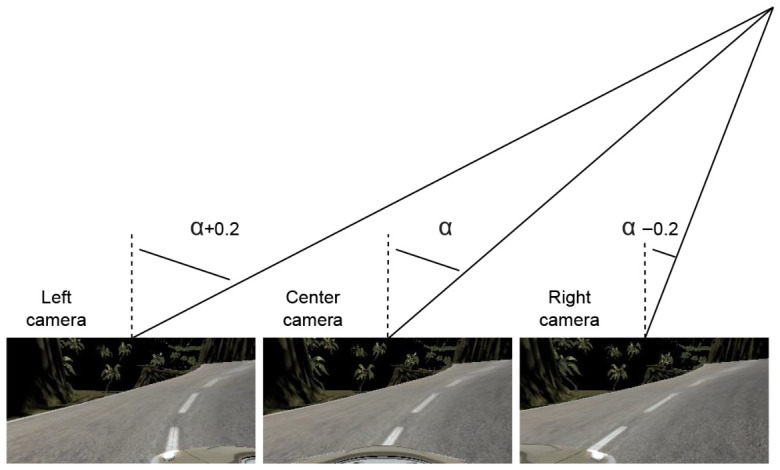
Correction of steering angle for lateral cameras. Modified from [11].

Lateral camera views can be seen as providing either a more pronounced or smoother representation of the curve captured by the central camera, as illustrated in Figure 4. However, since the steering angles recorded in the log file correspond to the central camera, the following procedure has been implemented to adjust the steering angle values for the lateral camera images:

- **Right camera**: The angle is corrected by subtracting 0.2 from the central camera value. This adjustment represents a sharper right turn.
- **Left camera**: The angle is corrected by adding 0.2 to the central camera value. This adjustment represents a sharper left turn.

**Figure 5:**
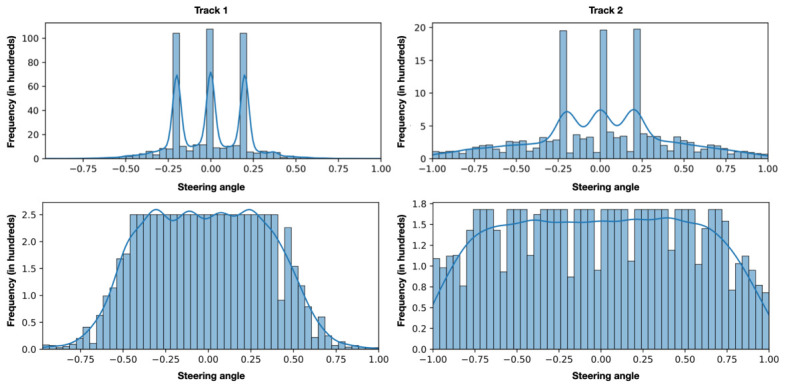
Histogram of the steering angle distribution after applying the correction for lateral cameras (**top**) and sample balancing (**bottom**).

Finally, since extreme values of the initial steering angle (−1 and +1) may result in data points outside the valid domain of the variable, values lower than −1 or higher than +1 are removed. The result is shown in the upper part of Figure 5.

##### Correction of the Steering Angle Distribution

The second stage of data preprocessing consists of balancing the steering angle distribution to achieve a more uniform distribution across both tracks. This step is essential to improve model generalization and to prevent excessive bias toward neutral angles. In an AD scenario, accurately predicting the most frequent steering angles is not sufficient; the vehicle must be able to predict any steering angle within the valid domain with the same precision to successfully navigate any curve.

Balancing the data ensures a uniform sampling of the model’s output domain, preventing biases during training. The following procedure describes how this balancing is achieved for each track:

1. **Interval division**: The range of steering angle values ([−1,1]) is divided into 50 equidistant intervals, grouping samples according to their angle values.
2. **Calculation of the maximum number of samples per interval**: A maximum sample limit per interval is set to ensure a uniform data distribution.
3. **Selection of samples per interval**: For each interval, samples are retained if they do not exceed the maximum limit; otherwise, excess samples are randomly removed.

This procedure guarantees uniformity in the data distribution within the representative range of each track. Specifically, for track 2, which contains sharper curves, the samples span the entire range of input values [−1, 1]. In the case of track 1, which has smoother curves, uniformity is ensured within the range [−0.45, 0.45]. The final distribution is shown in the lower part of Figure 5.

Additionally, this procedure has been used to reduce the data volume and achieve a similar data volume for both tracks, resulting in 7031 and 7042 samples for tracks 1 and 2, respectively. For each of the 50 intervals into which the steering angle is divided, track 1 has a maximum of 250 samples, while track 2 has a maximum of 168.

**Generation of Training and Validation Sets:** After distribution correction, the dataset is divided into training and test sets with an 80/20 split for each track. The focus is on comparing CL methodologies across reference models, all of which will be trained under the same conditions.
**Image Preprocessing:** The following steps outline the common preprocessing applied to the images (Figure 3). If a step is dataset-specific, it is explicitly stated.
**Image Resizing:** Images are resized to a uniform size to match the model architecture, which is inspired by NVIDIA’s PilotNet [34] (described later). This process reduces computational complexity and ensures consistency in model input.
**Data Augmentation for Training:** To increase the variability of the training data, variations in brightness, contrast, saturation, and tint are applied to images with random intensities. This data augmentation aims to enhance training robustness, improving generalization by enabling the model to recognize relevant features regardless of environmental variations, while also mitigating the risk of overfitting.  

The applied variations are as follows:

- **Brightness**: The global intensity of the image is multiplied by a random factor sampled from the interval [0.8, 1.2]. This results in brighter images (simulating a sunlit road) or darker images (simulating a shaded road).
- **Contrast**: The contrast is modified by multiplying the difference between the pixel values and their mean by a random factor from the interval [0.8, 1.2]. This simulates visual conditions with higher or lower definition between bright and dark areas.
- **Saturation**: The intensity of colors is multiplied by a random factor within the interval [0.8, 1.2]. This produces images with either duller or more vibrant colors, simulating different lighting conditions.
- **Tint**: The color hue is adjusted by adding a random offset within the interval [−0.1, 0.1]. This introduces slight changes in the color of the road or sky, simulating different times of day or weather conditions.

Although images are later converted to grayscale, applying saturation and tint modifications to the original color image introduces variations that influence luminance distribution after conversion, enriching the dataset and increasing visual diversity.

**Grayscale Conversion:** Images are converted to grayscale in order to reduce dimensionality and remove chromatic information, thereby simplifying data representation without compromising essential spatial information.

The transformation follows a standard formula for converting RGB images to grayscale, which weights the red (R), green (G), and blue (B) channels according to their perceived luminance contributions, as defined by the ITU-R BT.709 standard [35] (Equation (1)), used by default in the Pillow library ([36]).

(1) Y=0.2126·R+0.7152·G+0.0722·B

**Normalization:** Images are normalized within the range [0, 1] to facilitate model learning, as inputs are originally bounded within [0, 255].
**Data Encoding into Spikes:** As discussed in Section 3.1, there are multiple approaches to transform data into spike trains to feed SNNs. Although comparing the performance of different encoding schemes could provide valuable insight, such an analysis is beyond the scope of this work. Instead, a rate encoding approach is adopted, previously validated in related studies, using parameters that offer good training results for this architecture while maintaining a balance between accuracy and computational efficiency. The parameters used for encoding are as follows:

- **Number of timesteps (*S*) = 25**: This parameter determines the number of temporal steps available to represent an input value as spikes. It has been tested that 25 steps are sufficient to capture the dynamics of the available data without significantly increasing the computational burden during model training. As discussed in Section 3.1, each input image will be represented in 25 *S*. Increasing this number allows for more precise encoding, but substantially increases the volume of data to be processed.
- **Gain = 0.5**: This acts as a scaling factor that modulates the intensity of the input signal before converting it into spikes. In this specific case, where grayscale images are encoded into spikes using rate coding, higher pixel values correspond to higher firing rates. However, excessively high firing rates reduce network efficiency due to a loss of spike sparsity. Given the 256 possible grayscale values per pixel, the range of encoded firing rates can be reduced. Setting the gain (*G*) to 0.5 lowers the input intensity to prevent excessively high firing rates after encoding, ensuring a sparse signal representation and enhancing efficiency.

**Implications for sharp-turn sensitivity:** A concern in datasets with heavy neutral-angle bias is that the SNN’s firing threshold may suppress sensitivity to infrequent, large-magnitude steering commands. However, in this implementation, the output steering angle is extracted directly from the membrane potential of the single output LIF neuron rather than from spike counts or spike rates. This is a deliberate design choice: because the membrane potential is a continuous analog variable that integrates the input over time, it captures fine-grained gradations of the steering signal, including sharp-turn values near the extremes of the normalized range [−1,+1], without being subject to the quantization artifacts that would arise if the output were derived from discrete spike events. The gain parameter (G=0.5) acts exclusively on the input encoding layer, reducing firing rates in the convolutional feature maps to preserve sparsity; it does not constrain the output neuron’s membrane potential, which remains free to represent the full continuous range of the target variable. Together with the uniform sample balancing described above, this output decoding strategy ensures that the model is exposed to, and capable of representing, the full steering angle domain during both training and inference.
**Description of the Selected SNN Architecture:** As mentioned previously, this study is based on the implementation of a convolutional SNN derived from NVIDIA’s PilotNet architecture, designed for AD, with the purpose of predicting the steering angle of a vehicle from images captured by onboard cameras, one of the key requirements of this study. This architecture and its variants have been widely used in the context of the Udacity simulator, with demonstrated effectiveness in various studies (e.g., [11]).

One of PilotNet’s strengths is its simplicity and efficiency. It is a relatively compact architecture consisting of convolutional layers that extract spatial patterns, followed by fully connected layers that generate the final prediction. This structural simplicity makes it easy to adapt and implement Leaky Integrate-and-Fire (LIF) units, which are characteristic of SNNs.

Moreover, it provides a good balance between complexity and trainability. It is not excessively deep, which helps mitigate overfitting, particularly in moderate-sized datasets such as the one used in this project, and its compact structure enables efficient training.

The SNN implemented in this study differs from the original NVIDIA paper [34] in several key aspects. First, unlike the original model where input images are encoded using RGB channels, the images here retain their original size, but only the luminance component from the RGB is extracted and then encoded as spike trains to feed the model.

Furthermore, while the original network structure is preserved, the activation function of fully connected layers (ReLU) is replaced by LIF units, which are characteristic of SNNs, using the implementation available in snnTorch.

Thus, the network structure is as shown on Table 2.

The first five layers are convolutional, designed to extract spatial features from the input images, then the outputs are flattened and passed through a series of fully connected layers and, finally, output is reduced to a single LIF unit from which the membrane potential is extracted as the predicted steering angle. Both convolutional and fully connected layers follow the standard PyTorch [37] implementation, while snnTorch provides the LIF neuron implementation, seamlessly integrating into PyTorch as an activation function.

Unlike other adaptations of the PilotNet network, where dropout layers are introduced to improve generalization and prevent overfitting, the inherent sparsity of SNNs renders this unnecessary in our case.

### 3.4. Computational Complexity Analysis

To provide a rigorous foundation for understanding the energy-efficiency trade-offs in this study, we present a detailed analysis of computational complexity for the SNN architecture employed. This analysis draws on established methods from our prior work [2] and extends them to the CL context.

#### 3.4.1. Parameter Count and FLOPs Analysis

The SNN adaptation of PilotNet preserves all convolutional and fully connected weight tensors from the original CNN architecture. Spiking activations (LIF neurons) are inserted with zero additional learnable parameters, they introduce temporal dynamics through membrane potential evolution but do not add trainable weights. Therefore:

(2) $$\begin{array}{c} \begin{array}{c} \#{\mathrm{Params}}_{\mathrm{SNN}}=\#{\mathrm{Params}}_{\mathrm{CNN}}=252,219 \end{array} \end{array}$$

For floating-point operations (FLOPs), a CNN forward pass requires multiply-accumulate operations (MACs) determined by layer dimensions. For a convolutional layer:

(3) $$\begin{array}{c} \begin{array}{c} {\mathrm{MACs}}_{\mathrm{conv}}=H_{\mathrm{out}}\times W_{\mathrm{out}}\times C_{\mathrm{out}}\times \left(C_{\mathrm{in}}\times k_{h}\times k_{w}\right) \end{array} \end{array}$$

where $H_{\mathrm{out}},W_{\mathrm{out}}$ are output spatial dimensions, $C_{\mathrm{out}},C_{\mathrm{in}}$ are output/input channels, and $k_{h},k_{w}$ are kernel dimensions. Each MAC typically corresponds to 2 FLOPs (one multiply, one add).

For SNNs, the architecture is temporally unrolled over *S* timesteps. At each timestep, the same operations are repeated, leading to a linear scaling relationship:

(4) $$\begin{array}{c} \begin{array}{c} {\mathrm{FLOPs}}_{\mathrm{SNN}}\left(S\right)=S\times {\mathrm{FLOPs}}_{\mathrm{CNN}}\left(1\right) \end{array} \end{array}$$

Using the fvcore profiling library (as validated in [2]), we measured the following for PilotNet on input size 1×1×66×200 (see Table 3):

The near-perfect 25× and 50× scaling confirms the linear relationship. This establishes that:

(5) $$\begin{array}{c} \begin{array}{c} \frac{{\mathrm{FLOPs}}_{\mathrm{SNN}}\left(50\right)}{{\mathrm{FLOPs}}_{\mathrm{SNN}}\left(25\right)}=\frac{50}{25}=2 \end{array} \end{array}$$

#### 3.4.2. Spike Sparsity and Effective Computation

It is important to note that the measured FLOPs represent an upper bound on actual computation in SNNs. The nature of spiking neurons, which is driven by events, shows that operations in computation happen primarily in response to the occurrence of spikes; hence, the overall cost of computation is heavily shaped by spike frequency. The encoding analysis reported in [2] documented average spike rates of 35–45% of the theoretical maximum for rate encoding (S=25, G=0.5), 5–10% for delta encoding (θ=0.5), and approximately 4% for latency encoding (S=25, τ=5.0), where each neuron fires at most once per inference. This sparsity translates into computational savings through two complementary mechanisms. In the context of neuromorphic computing, neurons modify their states solely with the arrival of input spikes, which means a neuron without input does not involve itself in computational activity. Additionally, spiking neuron updates involve primarily integer additions and threshold comparisons rather than the floating-point multiply accumulate operations that dominate conventional neural network inference, making each individual operation cheaper on most hardware platforms.

We can estimate effective computational cost as:

(6) $$\begin{array}{c} \begin{array}{c} \mathrm{Effective}\;{\mathrm{FLOPs}}_{\mathrm{SNN}}\approx \mathrm{Theoretical}\;{\mathrm{FLOPs}}_{\mathrm{SNN}}\times \rho \times \alpha \end{array} \end{array}$$

where ρ is the spike rate (sparsity factor) and α is the operation cost ratio (binary vs. floating-point, typically 0.1–0.3 on conventional hardware, approaching 0.01 on neuromorphic chips).

For our rate-encoded baseline (S=25,ρ≈0.40) on conventional hardware (α≈0.20):

(7) $$\begin{array}{c} \begin{array}{c} \mathrm{Effective}\;\mathrm{FLOPs}\approx 580,768,550\times 0.40\times 0.20\approx 46,461,484 \end{array} \end{array}$$

This is only 2× the CNN’s FLOPs despite 25× temporal unrolling, explaining why measured energy consumption (Section 4) shows much less than 25× increase.

#### 3.4.3. Energy-FLOPs Relationship and Validation

To validate the FLOPs analysis against measured energy consumption, we correlate theoretical computation with CodeCarbon measurements. From [2] and this study’s baseline experiments (see Table 4):

The dramatic improvement in FLOPs-per-Wh for Delta encoding (100× better than CNN) despite identical theoretical FLOPs confirms that spike sparsity is the dominant factor in energy efficiency. Rate encoding’s poorer efficiency (2.5× worse than CNN) reflects its dense spike trains negating the benefits of event-driven computation.

#### 3.4.4. Implications for Continual Learning

In the context of CL, computational complexity analysis provides several relevant insights. Regarding per-epoch cost, the SNN’s temporal unrolling over S=25 means that each training step performs 25× more forward passes than a CNN, although sparse activation reduces the effective cost to approximately 2–3× for Delta encoding, as discussed above. The overhead introduced by each CL strategy differs substantially. The EWC requires computing the Fisher information matrix after Task 1 training, which adds one backward pass per training sample, plus a regularisation term evaluated at each subsequent update, resulting in a total overhead of approximately 5–10% per epoch. Rehearsal, on the contrary, increases the number of samples processed per epoch in proportion to the replay ratio: at 15% rehearsal, the effective dataset size grows by a factor of 1.15, directly scaling the computational cost by the same amount. However, the per-epoch overhead of rehearsal does not necessarily translate into higher total training cost. The results presented in Section 4 show that the rehearsal models converge in approximately 55 epochs on average, compared to 60 epochs for the naive model, so the faster convergence partially offsets the additional cost per epoch and obtain net energy savings over the entire training process.

#### 3.4.5. Scaling Analysis for Multi-Task Scenarios

For *n* sequential tasks, we can project total computational cost:

(8) $$\begin{array}{c} \begin{array}{c} \mathrm{Total}\;{\mathrm{FLOPs}}_{\mathrm{naive}}=n\times {\mathrm{FLOPs}}_{\mathrm{full}\;\mathrm{training}}=n\times E\times {\mathrm{FLOPs}}_{\mathrm{epoch}} \end{array} \end{array}$$

where *E* is epochs per task. For cumulative training:

(9) $$\begin{array}{c} \begin{array}{c} \mathrm{Total}\;{\mathrm{FLOPs}}_{\mathrm{cumulative}}=\sum_{i=1}^{n}E_{i}\times {\mathrm{FLOPs}}_{\mathrm{epoch}}\times i \end{array} \end{array}$$

For rehearsal with fixed buffer size *M*:

(10) $$\begin{array}{c} \begin{array}{c} \mathrm{Total}\;{\mathrm{FLOPs}}_{\mathrm{rehearsal}}=\sum_{i=1}^{n}E_{i}\times {\mathrm{FLOPs}}_{\mathrm{epoch}}\times \left(1+r\right) \end{array} \end{array}$$

where *r* is the replay ratio (constant). This shows rehearsal scales linearly O(n) while cumulative scales quadratically $O(n^{2})$.

This analysis establishes that SNNs offer computational advantages primarily through spike sparsity, which is maximized by Delta encoding. When combined with linear-scaling CL strategies like rehearsal, the approach provides sustainable scalability for multi-task learning scenarios, a critical requirement for real-world AD systems that must continuously adapt to new environments.

### 3.5. Problem Definition and CL Scenario

The scenario has been defined according to several constraints that are fundamental to CL and will determine the evaluation of the implemented methodologies. These constraints are as follows:

1. **Domain-Incremental Scenario**
  The selected scenario corresponds to a domain-incremental setting, where the output domain of successive tasks remains the same (predicting the steering angle, in this case), but the input data domain differs.
  In this specific study, each task represents a different track and the model must generalize across different tracks (or visual environments).
2. **Data Availability: Two Sequential Tasks**
  The data is introduced sequentially, structured as two distinct tasks: Task 1 consists of data from Track 1, followed by Task 2, which includes data from Track 2. Simultaneous access to data from both tasks during training is not allowed, which simulates the typical constraint in CL.
3. **Task-Agnostic Approach**
  The model receives no explicit knowledge about which task it is facing at inference time, an approach known as task-agnostic learning.
  The evaluation will be performed on both tasks with the objective of identifying the model that achieves the best overall performance.

**Figure 6:**
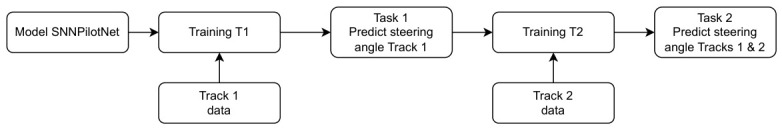
Task sequencing.

Figure 6 shows the task sequencing in the CL scenario described above.

### 3.6. Reference Models

**Figure 7:**
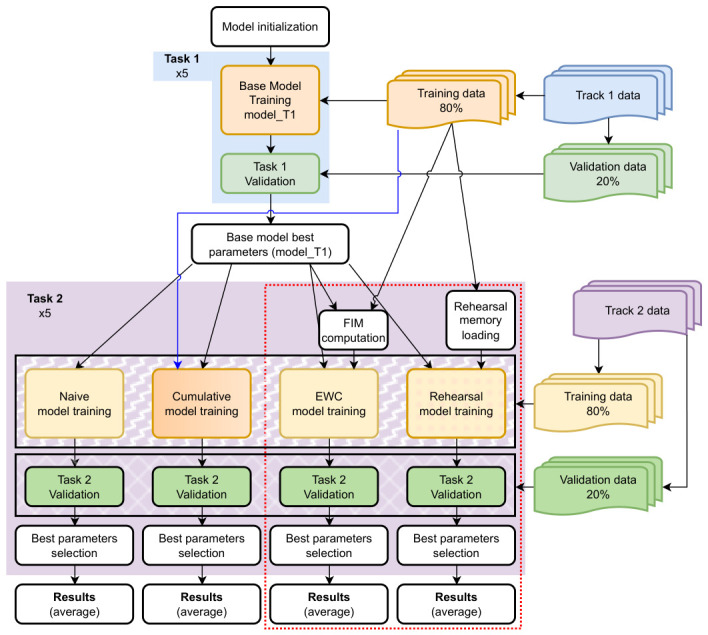
Summary of the training and evaluation process of the models developed in this study. The red box highlights models implementing CL strategies.

To establish valid references that allow for the performance comparison of the methodologies implemented to mitigate catastrophic forgetting, three reference models are defined. Figure 7 illustrates the sequence of steps for training and validation of the models developed in this study.

#### 3.6.1. Baseline Model (Model_T1): Task 1

To establish a common reference point for comparing different models within the CL scenario, a baseline model is defined. This model is trained from scratch using data from Track 1. Thus, it represents Task 1 within the CL framework and serves as the starting point for subsequent experiments.

**Randomness Control:** Since both model initialization and training incorporate multiple sources of randomness, such as weight initialization and mini-batch sampling, a fixed random seed is set. Additionally, the training process is repeated five times to:

1. Characterize the inherent variability in the training results.
2. Establish the mean and standard deviation of the model performance, improving the robustness of the conclusions.

Once the repetitions are completed, the model that yields the best validation performance in Track 1 is selected. Track 2 data are not used to train the model at this stage, though the model will be evaluated on this track as well for future comparison purposes.

**Loss Function:** The chosen loss function for the optimization process is the Mean Squared Error (MSE), which calculates the mean squared differences between the predicted and the target values. This metric is widely used in regression problems and heavily penalizes large errors, thus improving the accuracy of the model during training.
**Training Hyperparameters:** To evaluate the effect of different hyperparameters on the performance of the model, a hyperparameter search was carried out using the Optuna library ([38]). The search space and the five best results are presented in Table 5. A total of 100 experiments were performed within the search space, along with an additional fixed experiment (highlighted in purple in Table 5). In all cases, the model was trained for 15 epochs using Track 1 data.

The selected training parameters were chosen to balance performance, convergence stability and consistency with previous works. Ultimately, the parameters highlighted in blue were selected, as they provided the best trade-off between performance (MSE) and efficiency (in terms of experiment duration).

These parameters will remain fixed across all subsequent experiments to ensure a fair comparison between models. The training process and the establishment of the initial reference model (model_T1) is illustrated in Figure 7.

#### 3.6.2. Naive Model: Sequential Training Without CL Strategies

The naive model is defined as a model trained sequentially on Tasks 1 and 2 without implementing any technique to mitigate catastrophic forgetting. This model serves as a reference for both computational cost and efficiency in sequential training, as well as for quantifying the degree of forgetting on Task 1 after training with Task 2 data. The results obtained establish a baseline to compare and validate subsequent CL methodologies.

To establish this reference, the following sequence is followed (Figure 7):

- **Initial Preparation**: The training starts with model_T1, which consists of the model weights obtained after training on Track 1. This model has already been selected as the optimal point for Task 1.
- **Sequential Training**: Without applying any strategy to mitigate forgetting, the training continues on Task 2 (Track 2). The training process continues until a maximum of 60 total epochs (sum of training epochs on both tasks) is reached.
- **Best Model Selection**: The model is evaluated on the validation set of Task 2 after each epoch. The best model is selected based on the best validation loss for Track 2.
- **Performance Evaluation**: After training is completed, model performance is assessed based on loss values for the validation sets of:
  - **Track 1 (Task 1)**: To quantify catastrophic forgetting by comparing the current MSE with that of model_T1.
  - **Track 2 (Task 2)**: To characterize the model’s ability to learn the new task.

As before, the sequence is repeated five times to constrain variability in the results.

#### 3.6.3. Cumulative Model

The cumulative model is defined as a model that is trained for each task using all available data from previous tasks along with the data from the current task. This means that it does not suffer from the limitations of restricted access to past data and thus breaks the constraints defined in this CL scenario. Nevertheless, it provides an essential theoretical reference of the ideal performance achieved by a model without constraints while also quantifying the computational cost of an intensive data accumulation strategy.

To establish this reference, the sequence shown in Figure 7 is followed:

- **Initial Preparation**: As in the previous case, the training starts with model_T1.
- **Cumulative Sequential Training**: Without applying any strategy to limit task forgetting, the training continues using the combined data from both Task 1 and Task 2 (as indicated by the blue arrow in Figure 7). The training is carried out until a total of 60 epochs is reached.
- **Best Model Selection**: The model that achieves the best validation performance on the current task (Task 2) is selected.
- **Performance Evaluation**: The performance of the model is assessed on the validation sets of all tasks to determine the maximum performance that the model can achieve.

These results allow us to:

- Establish theoretical upper bounds on performance, assuming unlimited access to data.
- Define the computational and energy costs that no CL implementation should exceed.
- Quantify the potential improvements brought about by strategies designed to mitigate catastrophic forgetting.

### 3.7. Implementation of Continual Learning Strategies

This section describes the implementation of two strategies proposed to address the CL problem and mitigate the effects of catastrophic forgetting: Elastic Weight Consolidation (EWC) and Rehearsal. These techniques are compared with the previously defined reference models (naive and cumulative) to analyze their effectiveness and the improvements they bring to the sequential learning scenario.

In the domain of CL, both strategies are widely used. The first strategy, EWC, offers minimal computational overhead in comparison to a naive model. The second strategy, Rehearsal, is the most recommended when the application environment allows the storage of past data for replay.

These implementations follow the training conditions defined for the reference models in the preceding section (see Figure 7, red box). This ensures that the impact of each strategy is quantified in terms of performance, catastrophic forgetting, and computational efficiency. The technical and methodological details of each approach are described below.

#### 3.7.1. Elastic Weight Consolidation (EWC)

EWC was introduced as a regularization-based method to mitigate catastrophic forgetting. The core idea behind EWC is to selectively constrain the parameters that are important for previously learned tasks while allowing other parameters to adapt to new tasks. This is achieved by estimating the importance of each parameter using the Fisher information matrix (FIM), which measures the sensitivity of the model’s performance to changes in each parameter [39,40].

**Theoretical Basis and Implementation:** EWC is inspired by the biological behavior of synaptic plasticity, where certain neural connections remain stable to preserve critical information, while others remain flexible to incorporate new learning.  

The goal of EWC is to consolidate important weights from previous tasks by applying a regularization-based penalty, preventing these weights from being significantly altered during the learning of new tasks.

Conceptually, the method is based on the premise that there is no single optimal weight configuration for a given task, but rather a set of valid solutions. Given two or more tasks, their respective solution sets may overlap or be disjoint. The regularization (or penalty) applied to the loss function during training of the next task aims to guide the gradients toward an optimal solution common to all tasks (if solution sets overlap) or toward a compromise solution (if they are disjoint).

**Figure 8:**
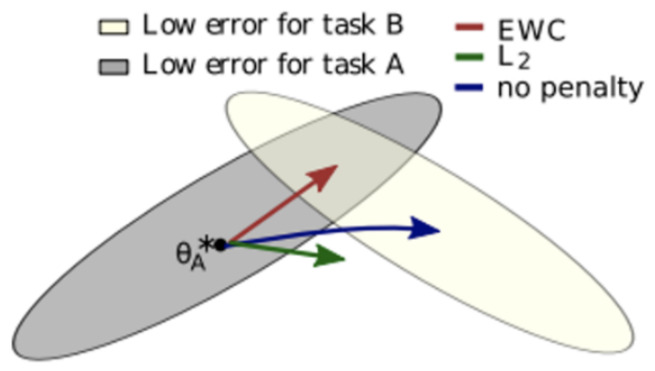
Effect of EWC regularization. Adapted from [41].

Figure 8 graphically represents a scenario in which two tasks (A and B) have respective solution sets (gray and ochre, respectively) that overlap at the top. The sequential training of tasks A and B leads to a migration of the network weights, as indicated by the blue arrow, shifting from the center of the optimal solution set for Task A to the center of the optimal solution set for Task B. This results in catastrophic forgetting of Task A, despite the model performing correctly on Task B. The red arrow indicates, on the other hand, the evolution of the network weights during Task B training with EWC regularization. In this case, the weights migrate to an optimal value for both Task A and Task B (at the intersection of the solution sets), enabling effective learning of Task B while preserving knowledge of Task A.

The additional term applied to the loss function is based on the Fisher Information Matrix, which estimates the importance of each weight based on its contribution to the previous task.

The adjusted loss function for EWC is shown in Equation (11):

(11) $$L_{\mathrm{EWC}}=L_{\mathrm{new}\;\mathrm{task}}+\lambda \sum_{i}F_{i}{\left(\theta_{i}-\theta_{i}^{*}\right)}^{2}$$

where

- $L_{\mathrm{new}\;\mathrm{task}}$ is the loss associated with the current task being trained.
- λ is the hyperparameter that controls the intensity of the penalty applied to the weights that are important for previous tasks.
- $F_{i}$ is the value of the Fisher Information Matrix for the weight *i*, estimating its importance relative to previous tasks.
- $\theta_{i}$ is the current value of the weight *i* during the training of the new task.
- $\theta_{i}^{*}$ is the value of the weight *i* learned from previous tasks.

Two important aspects for the application of EWC are:

1. **Estimating Weight Importance**: This is done by computing the Fisher Information Matrix after completing training on each task.
2. **Tuning the λ Parameter**: This ultimately controls the plasticity–stability balance of the model.

The FIM is calculated from the derivatives of the probability function with respect to the weights of the model. However, computing the full matrix is computationally expensive. To efficiently implement Fisher matrix estimation, the diagonal approximation is commonly used. In this approach, only diagonal elements of the FIM are considered, assuming statistical independence among the weights. Although this assumption may introduce errors in weight importance estimation, empirical results show that it is generally effective.

**Validity of the diagonal FIM approximation in SNN-BPTT training:** A natural question arises as to whether the diagonal approximation remains valid when EWC is applied to SNNs trained via BPTT with surrogate gradients, given that temporal unrolling across *S* could in principle introduce cross-parameter correlations not captured by the diagonal. However, in the implementation adopted here, the loss gradient with respect to each weight is computed as the sum of contributions accumulated over the fixed temporal window of S=25. Because the model does not maintain separate weight copies per timestep (all *S* share the same parameter tensor) the effective parameter space remains identical in dimensionality to a standard ANN, and the diagonal FIM computed at convergence reflects per-weight sensitivity in this temporally averaged sense. The temporal dependencies are implicitly encoded in the gradient magnitudes themselves rather than in off-diagonal weight correlations. This treatment is consistent with the EWC implementations in [42,43], which apply the diagonal approximation to temporally unrolled networks without reporting degradation attributable to the approximation. Empirically, the results presented in Section 4 confirm that the diagonal FIM provides a useful importance signal: the FR is reduced relative to the naïve model across all tested λ values, demonstrating practical effectiveness even if theoretical optimality cannot be guaranteed. Extensions such as Kronecker-factored EWC (K-FAC) [44] could capture richer weight correlations and are identified as a direction for future work (Section 5).

Moreover, the computation can be further simplified in this case, as the loss function can be used instead, avoiding explicit probability computation.

The diagonal approximation of the Fisher matrix is computed as shown in Equation (12):

(12) $$F_{ii}=E\left[{\left(\left(f\left(x;\theta \right)-y\right)\cdot \frac{\partial f(x;\theta )}{\partial \theta_{i}}\right)}^{2}\right]$$

where

- $F_{ii}$ is the diagonal element of the Fisher matrix.
- E denotes the expectation.
- f(x;θ)−y represents the prediction error.
- $\frac{\partial f(x;\theta )}{\partial \theta_{i}}$ is the gradient of the function with respect to $\theta_{i}$.

The pseudocode in Algorithms 1 and 2 details the implementation used in this study.

**Description of the Experiments:** To evaluate the effect of regularization through EWC, the training sequence for Task 2 will be repeated as described for the reference models, with the following modifications (also indicated in Figure 7, red box, EWC models):

- **Initial Preparation**: Training starts with the baseline model trained on Task 1 (model_T1), as previously described.
- **Sequential Training**: From model_T1, the parameters that influence the gradient of the loss function are saved and the Fisher matrix is computed for these parameters. Training then continues with Task 2 (Track 2) data, applying the EWC regularization. The process continues until a maximum of 60 total epochs is reached.
- **Best Model Selection**: The parameters corresponding to the epoch with the best validation results in Track 2 (measured in terms of loss) are selected as the best model.
- **Performance Evaluation**: After training, the model’s performance is evaluated based on the loss values of the validation sets of Track 1 and Track 2.

**Algorithm 1** Training with Elastic Weight Consolidation (EWC) Task 1**Require:** Initialized model parameters *P*, dataset D1 (Task 1), dataset D2 (Task 2), regularization coefficient λ, MSE loss function loss_mse**Ensure:** Model *P* trained on Task 2 with EWC regularization  1:  **Task 1: Initial Training**  2:  Train the model *P* on dataset D1 until convergence:  3:      P_final←train(D1,loss_mse)  4:  Select only the parameters that require updates:  5:      Initialize param_set ←∅  6:  **for** each parameter *p* in P_final **do**  7:        **if** *p* has non-zero gradients **then**  8:              Add *p* and its current value to param_set  9:        **end if**10:  **end for**11:  Compute the diagonal Fisher information matrix:12:      Initialize F←{0foreachp∈param_set}13:  **for** each batch (x,y)∈D1 **do**14:        Zero model gradients15:        y_pred←model(x,P_final)16:        loss←loss_mse(y,y_pred)17:        Backpropagate the loss18:        **for** each p∈param_set **do**19:              $g_{p}\leftarrow \mathrm{current}\_\mathrm{gradient}\left(p\right)$20:              $F\left[p\right]\leftarrow F\left[p\right]+g_{p}^{2}$21:        **end for**22:  **end for**23:  Normalize F[p]←F[p]/|D1|24:  Save param_set and Fisher matrix:25:  params_task1←param_set26:  fisher_task1←F

**Algorithm 2** Training with Elastic Weight Consolidation (EWC) Task 2  1:  **Task 2: Training with EWC**  2:  **for** each batch (x,y)∈D2 **do**  3:        Compute current task loss:  4:        $L_{task2}\leftarrow \mathrm{loss}\_\mathrm{mse}\left(\mathrm{model}\left(x,P\right),y\right)$  5:        Compute EWC regularization loss:  6:        $L_{EWC}\leftarrow 0$  7:        **for** each parameter p∈params_task1 **do**  8:              $reg_{p}\leftarrow \mathrm{fisher}\_\mathrm{task}1\left[p\right]\cdot {\left(p\_current\left[p\right]-\mathrm{params}\_\mathrm{task}1\left[p\right]\right)}^{2}$  9:              $L_{EWC}\leftarrow L_{EWC}+reg_{p}$10:        **end for**11:        Combine total loss:12:        $L_{batch}\leftarrow L_{task2}+\lambda \cdot L_{EWC}$13:        Update model parameters using $L_{batch}$14:  **end for**15:  **return** Final model *P* trained with EWC

As previously stated, this sequence will be repeated five times to characterize the variability of the results. Furthermore, since the optimal value of λ to effectively balance stability and plasticity is initially unknown, training will be conducted for different values of λ.

An initial trial tested five λ values uniformly distributed in a logarithmic progression between $10^{9}$ and $10^{15}$. After analyzing the results, the search range was refined, and training was repeated with λ values uniformly distributed between $10^{9}$ and $10^{12}$.

#### 3.7.2. Rehearsal Methodology

As a memory-based approach, Rehearsal methods are crucial in CL as they help maintain performance on previously learned tasks while adapting to new data. These methods typically involve storing a subset of past data and using it in conjunction with new data during training. The effectiveness of rehearsal methodologies can be enhanced by focusing on inter-task relationships, computational efficiency, sample selection, and memory management.

**Theoretical Basis and Implementation:** Unlike techniques such as EWC, which rely on weight penalties, Rehearsal stores a subset of representative examples from previous tasks in a memory buffer. During the training of new tasks, these examples are revisited to ensure that the model retains the knowledge acquired previously. Key aspects of the implementation include:

- The buffer examples are mixed with the new task data to create mini-batches during training.
- The total loss function is computed using both data sources, ensuring that the model learns the new task while retaining previous information.

The total loss function for Rehearsal is defined as follows (Equation (13)):

(13) $$L_{\mathrm{total}}=L_{\mathrm{new}}\left(D_{\mathrm{new}},\theta \right)+L_{\mathrm{memory}}\left(M,\theta \right)$$

where

- $L_{\mathrm{total}}$ is the total loss to be optimized during training.
- $L_{\mathrm{new}}\left(D_{\mathrm{new}},\theta \right)$ is the loss associated with the new task data $D_{\mathrm{new}}$ given the model parameters θ.
- $L_{\mathrm{memory}}\left(M,\theta \right)$ is the loss associated with the memory buffer examples *M* given the model parameters θ.

**Figure 9:**
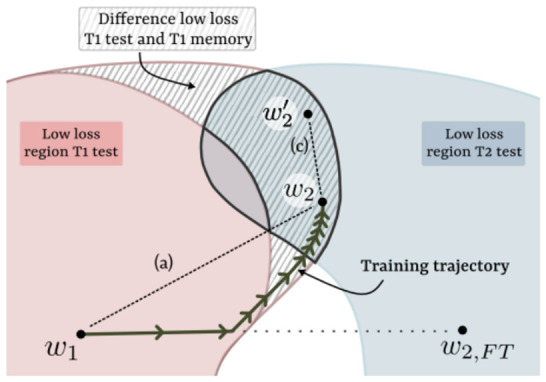
Effect of rehearsal on the gradient update trajectory. Adapted from [45].

As shown in Figure 9, incorporating previous task data during new task training influences the weight update trajectory, steering it toward a common low loss solution for all tasks. Similarly to EWC, the objective is to find a weight configuration that lies within the minimal loss region for all tasks (two in our case), but the mechanism differs from EWC.

Since this strategy increases the number of training samples, it directly impacts model efficiency and training computational cost, not only due to the memory required to store past data but also due to the increased volume of processed data. Therefore, in applying this method, an optimal trade-off must be found between model performance and increased computational cost. This involves determining the minimum required number of past task samples that still effectively represent that task distribution.

In fact, the cumulative model presented above can be interpreted as an extreme case of this strategy, where the memory buffer contains all previous task data.

**Implementation Considerations:** To implement the Rehearsal strategy, two key aspects must be defined:

1. **Buffer Size**: This directly influences the number of available samples for Rehearsal, as well as the process’s memory consumption.
2. **Sample Selection Strategy**: This determines which samples from Task 1 are stored in memory. Selection strategies can be:
  - **Random Selection**: The samples are randomly chosen from the Task 1 dataset.
  - **Importance-Based Selection**: Samples are selected based on their influence on weight updates or gradient variations.

From a computational cost perspective, random selection is the most efficient, as it does not require computing sample importance. In addition, it is a well-established strategy in the literature and typically yields good results. Therefore, this strategy has been implemented in this study.

Another important factor is the proportion of training samples taken from memory during training. It must be determined whether these samples are selected with or without replacement.

The Rehearsal memory implemented in this study allows for the selection of both buffer size and the proportion of memory samples relative to batch size. For each epoch, the samples are randomly selected from the buffer without replacement, ensuring that (Equation (14)):

(14) $$r_{\mathrm{repetition}}<r_{\max}=\frac{M}{M+D_{\mathrm{task}2}}$$

where

- $r_{\mathrm{repetition}}$ is the chosen repetition rate.
- $r_{\max}$ is the maximum achievable rate without replacement.
- *M* is the buffer size.
- $D_{\mathrm{task}2}$ is the dataset size for Task 2.

The complete implementation is presented in the pseudocode in Algorithms 3 and 4.

**Description of the Experiments:** To evaluate the effect of Rehearsal memory, the training sequence for Task 2 will be repeated with the following modifications (Figure 7, red box, Rehearsal models):

- **Initial Preparation**: Training starts with model_T1, as previously described.
- **Sequential Training**: The Rehearsal buffer is loaded with data from Task 1, and training continues on Task 2 (Track 2) data, incorporating the memory buffer. The process continues until a maximum of 60 total epochs is reached.
- **Best Model Selection**: The model’s performance is evaluated on the validation set of Task 2 after each epoch. The parameters corresponding to the epoch with the best validation results on Track 2 (measured in terms of loss) are selected.
- **Performance Evaluation**: Once training is complete, the best parameters are chosen based on the loss values in the validation sets of Tracks 1 and 2.

**Algorithm 3** Training with Rehearsal Task 1**Require:** Initialized model parameters *P*, datasets D1 and D2, loss function loss_mse,  1:  maximum buffer size buffer_size (i.e., mida_M),  2:  empty buffer *M*, replay ratio replay_ratio∈(0,1), total batch size batch_size**Ensure:** Model *P* trained on Task 2 with rehearsal, and final buffer *M*  3:  **Task 1: Initial Training**  4:  Train the model *P* on D1 until convergence:  5:      P_final←train(D1,loss_mse)  6:  Populate buffer *M* with samples from D1:  7:  Initialize total_seen←0  8:  **for** each sample (x,y)∈D1 **do**  9:        total_seen←total_seen+110:        **if** buffer *M* has fewer than buffer_size elements **then**11:              Add (x,y) to buffer *M*12:        **else**13:              Randomly select index k∼U(0,total_seen−1)14:              **if** k<buffer_size **then**15:                    Replace the *k*-th item in buffer *M* with (x,y)16:              **end if**17:        **end if**18:  **end if**

**Algorithm 4** Training with Rehearsal Task 2  1:  **Task 2: Training with Rehearsal**  2:  Compute batch sizes for rehearsal and new data:  3:  buffer_batch_size←⌊replay_ratio×batch_size⌋  4:  task_batch_size←batch_size−buffer_batch_size  5:  Verify replay ratio feasibility:  6:  $\max\_\mathrm{replay}\_\mathrm{ratio}\leftarrow \frac{\left|M\right|}{\left|M|+|D2\right|}$  7:  **if** 
replay_ratio>max_replay_ratio 
**then**  8:       Adjust replay_ratio←max_replay_ratio to avoid excessive repetition  9:  **end if**10:  Train the model *P* using combined batches:11:  **for** each training step **do**12:        Select task_batch_size unique samples from D2: D2_selected13:        Select buffer_batch_size unique samples from *M*: M_selected14:        Combine both sets to create batch:15:            batch_inputs,batch_labels←concat(D2_selected,M_selected)16:        Compute loss:17:            $L_{batch}\leftarrow \mathrm{loss}\_\mathrm{mse}\left(\mathrm{model}\left(\mathrm{batch}\_\mathrm{inputs}\right),\mathrm{batch}\_\mathrm{labels}\right)$18:        Backpropagate and update parameters:19:            $L_{batch}.backward\left(\right)$20:            Update model *P* using optimizer21:  **end for**22:  **return** Final model parameters *P* and buffer *M*

As before, this sequence is repeated five times to characterize the variability of the results. Furthermore, to test the effect of the Rehearsal buffer, its size is set to ensure that memory samples constitute 25% of the training data, corresponding to a buffer size of 1200 samples. The training is then repeated with repetition rates of 10%, 15%, 20%, and 25%.

### 3.8. Physical Neuromorphic Deployment Platform

The experiments reported in this work are executed on a conventional computing platform using PyTorch 2.11.0 and snnTorch 0.9.4, with energy consumption tracked via CodeCarbon [3]. However, the choice of SNN architecture and the emphasis on energy efficiency are deliberately aligned with the constraints of physical neuromorphic hardware, where the efficiency gains described in the computational complexity analysis (Section 3.4) are fully realized rather than approximated.

To this end, a companion deployment study [46] validated a low-cost, low-power physical platform consisting of a Raspberry Pi 5 (RPI5) paired with the BrainChip Akida PCIe accelerator (Akida board). This platform is directly relevant to the present work because it demonstrates the physical feasibility of deploying CNN-based models of the same class and complexity as those trained here onto dedicated neuromorphic hardware operating at under 10 W.

#### 3.8.1. Hardware Description

The RPI5 acts as the host machine. It integrates a quad-core ARM Cortex-A76 CPU, up to 8 GB of RAM, and a PCIe interface that allows for direct connection to the Akida board. Its 64-bit Debian-based Linux OS provides full compatibility with Python (3.11 Version) and TensorFlow, and its compact form factor makes it suitable for on-vehicle or edge deployment without GPU infrastructure.

The Akida board is a neuromorphic NPU that executes quantized neural network models using event-driven, asynchronous computation. Rather than processing entire feature maps at regular clock intervals, the chip triggers operations only in response to spike events, which significantly reduces both power and latency. The chip supports Conv2D, Dense, BatchNorm, Flatten, and ReLU operations, covering all the layer types present in the PilotNet-derived architecture used in this study (Table 2). The measured inference energy on this platform lies in the range of 10–30 μJ per sample, with latencies below 1 ms [46], representing several orders of magnitude improvement over CPU-based inference on the same board. The BrainChip Akida platform was selected in preference to alternatives such as Intel Loihi because it is commercially available without academic partnership requirements, ships with a well-documented Python SDK, and is directly compatible with TensorFlow/Keras-trained models. These properties made it the only platform compatible with the full model conversion workflow used in this study.

#### 3.8.2. Software Pipeline and Sensor Integration

On the software side, the deployment workflow described in [46] begins with training a model in TensorFlow 2.x using Quantization-Aware Training (QAT) via the TensorFlow Model Optimization Toolkit. QAT constrains weights and activations to 4–8 bit fixed-point precision during training by inserting fake quantization nodes into the computational graph, while allowing gradients to flow in full precision via the Straight-Through Estimator. This step is necessary because the Akida hardware executes models exclusively in low-bitwidth integer arithmetic, and post-training quantization alone produces significant accuracy degradation in compact regression models of this kind.

Once trained, the model is exported in HDF5 format and converted into Akida’s binary format through the Akida Python SDK. The SDK maps the supported layer types into spike, compatible operators optimized for event-driven processing and provides profiling utilities for real-time reporting of layer-wise latency, power draw, memory usage, and spike counts.

From a sensing perspective, the RPI5-Akida platform interfaces directly with camera modules, enabling a continuous pipeline from image capture to neuromorphic inference to actuation output. This pipeline architecture mirrors the one evaluated here in simulation: a front-facing camera supplies frames that are preprocessed (resized to 66×200 pixels, converted to grayscale) and forwarded to the network, which outputs a steering angle prediction from the membrane potential of the output neuron. The physical realization of this pipeline in [46] confirms that the sensor-to-inference latency budget is compatible with real-time AD constraints.

#### 3.8.3. Distributed Edge Operation

Beyond single-node inference, the platform supports distributed deployment through lightweight communication protocols. MQTT enables low-latency publish/subscribe broadcasting of inference outputs across a network of nodes, with measured end-to-end delivery times of approximately 6.2 ms (σ=1.1 ms) over a local area network [46]. UDP-based V2X broadcasting and HTTP-based federated model synchronization are also demonstrated, allowing multiple RPI5-Akida nodes to share inference results or updated model weights without cloud dependency. This distributed capability is particularly relevant to the CL scenario explored in this paper: in a real-world multi-environment driving setup, each vehicle or edge node could update its SNN model incrementally as it encounters new driving domains, then share the updated weights with other nodes via the federated synchronization protocol, realizing a physically grounded version of the sequential task learning evaluated here in simulation.

## 4. Results and Discussion

This section presents the results obtained during the training and evaluation of the models. We analyze predictive performance, catastrophic forgetting, backward transfer, computational cost and sustainability, and finally the implications and limitations of the study.

- **Mean Squared Error (MSE)**: This metric is used during training to assess the model performance and feed the optimizer. Lower MSE values indicate better predictive capacity. A high MSE suggests that the model’s predictions deviate significantly from the ground truth, indicating poor pattern recognition. Taking the square root of the MSE provides an estimate of the prediction bias in normalized degrees.
- **Forgetting Ratio (FR)**: Evaluates the model’s loss of knowledge on the first task after training on the second task. It is computed as
  $$FR=\frac{T_{1}^{2}-T_{1}^{1}}{T_{1}^{1}}\cdot 100$$
  where $T_{1}^{1}$ is the loss on the validation set of Task 1 after Task 1 training is complete, and $T_{1}^{2}$ is the same loss after Task 2 training. It is a measure of the stability of the model.
- **Learning Ratio (LR)**: Defined with respect to the loss values of the naïve reference model:
  $$LR=\frac{T_{2}^{\mathrm{variant}}-T_{2}^{\mathrm{naive}}}{T_{2}^{\mathrm{naive}}}\cdot 100$$
  where $T_{2}^{\mathrm{naive}}$ is the validation loss on Task 2 for the naïve model, and $T_{2}^{\mathrm{variant}}$ is the same metric for each variant analyzed. This measures the model’s plasticity compared to a non-CL approach.
- **Backward Transfer Learning**: Measures the impact of learning new tasks on the performance of previous ones ([30]). In this case, a negative FR indicates the presence of backward transfer, as the model improves its performance on Task 1 during Task 2 training.
- **Training Time**: Interpreted as a measure of model efficiency. While absolute values depend on the hardware, the relevance lies in comparing models executed under the same conditions.
- **Energy Consumption and CO_2_ Emissions**: Using the CodeCarbon library [3], energy consumption (in Wh) and equivalent CO_2_ emissions are tracked during model training. Energy consumption is the main hardware-agnostic sustainability metric reported in this work. CO_2_ emissions are reported as a secondary contextualized indicator derived from the carbon intensity of the local electrical grid at the time of experimentation (Germany, ≈400 gCO_2_/kWh, as reported by CodeCarbon via the Electricity Maps API [4]). These figures should not be interpreted as universal values, since grid carbon intensity varies substantially by region and time of day [4]. Both metrics gain significance primarily through relative comparison across models evaluated under identical conditions.

In the following sections, results for the most relevant models are discussed.

### 4.1. Reference Models

**Figure 10:**
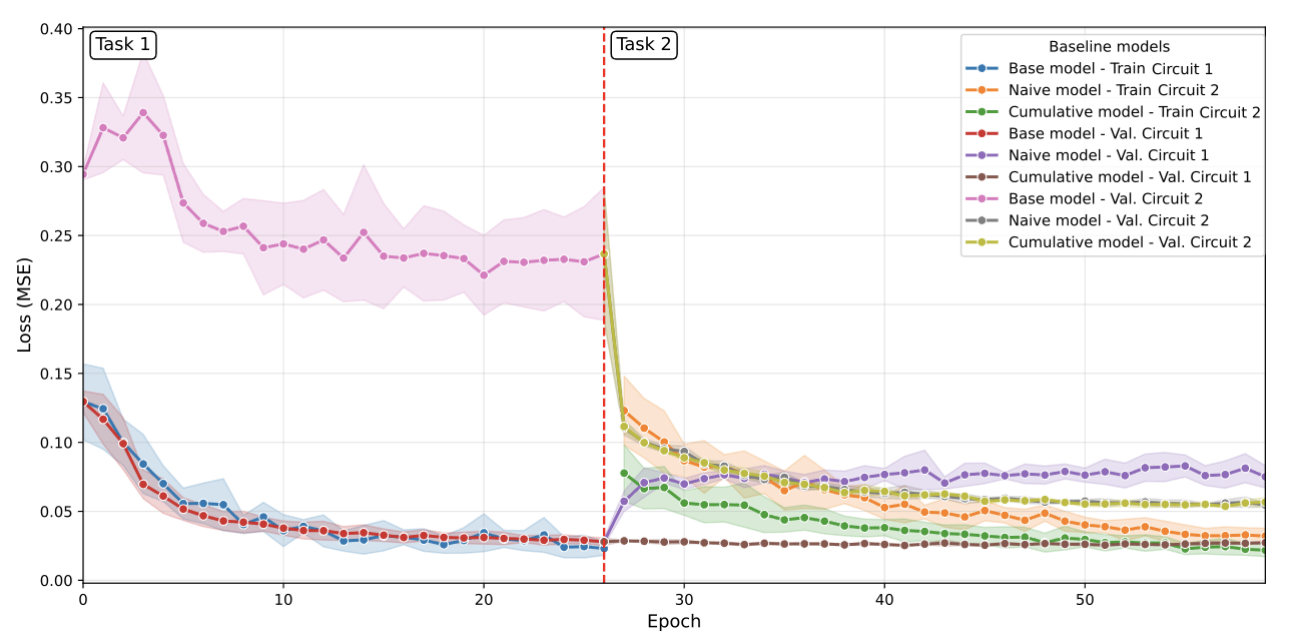
Training and loss curves for the reference models.

Figure 10 shows the training and loss curves for the reference models. The horizontal axis represents the epochs during training, and the vertical axis shows the value of the loss function (MSE). Lower loss values indicate better model performance.

The red vertical line marks the end of Task 1 (training on Track 1 data) and the start of Task 2 (training on Track 2 data). The position of the line corresponds to the epoch at which the baseline model achieves the best validation loss on Track 1. All Task 2 models are initialized with the parameters of the baseline model at this epoch (i.e., Task 1 training is common to all models).

**Figure 11:**
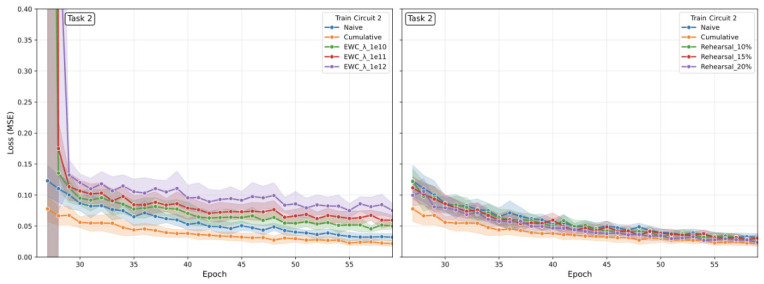
Training curves for EWC models (**left**) and rehearsal memory models (**right**).

**Figure 12:**
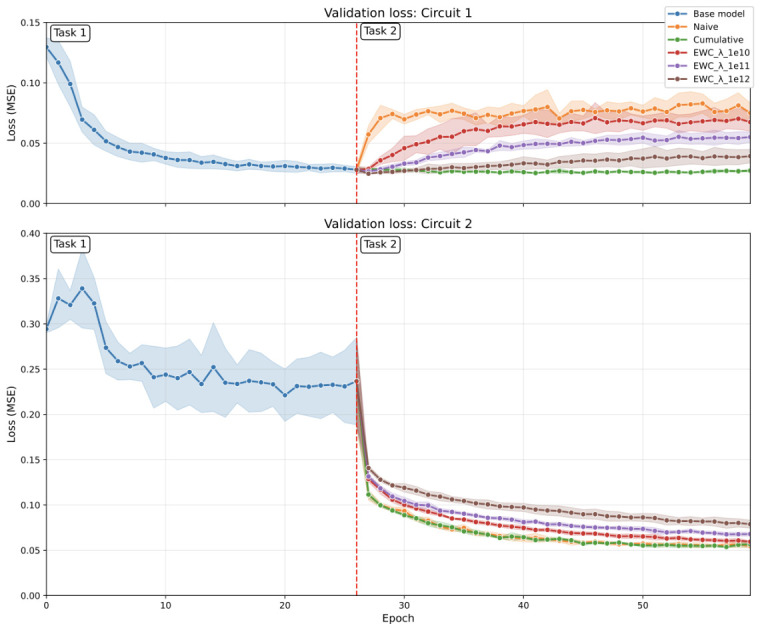
Validation loss curves for Tracks 1 (**top**) and 2 (**bottom**) for EWC models.

**Figure 13:**
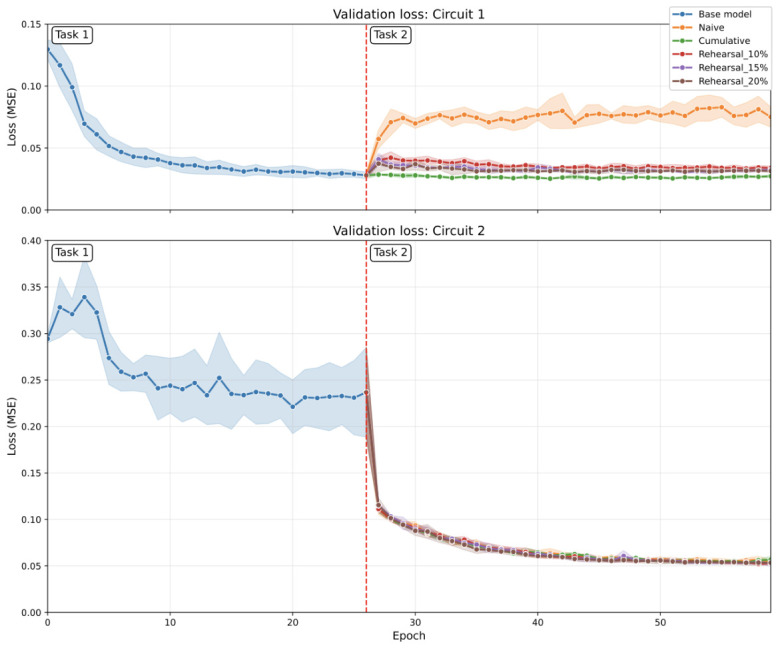
Validation loss curves for Tracks 1 (**top**) and 2 (**bottom**) for rehearsal models.

The curves represent the mean loss value across the five executions of each training run, while the shaded area indicates the standard deviation. Unless otherwise specified, Figure 11, Figure 12 and Figure 13 in this work follow the same visualization format.

The baseline model trained exclusively on Task 1 (Track 1) serves as the foundation, while the naive and cumulative models extend the evaluation to Task 2 (Track 2). Their evolution reveals that the naive model suffers from catastrophic forgetting of Task 1, whereas the cumulative model achieves the best overall performance by jointly using both datasets, even benefiting from backward transfer learning (improvements in Task 1 after Task 2 training).

#### 4.1.1. Baseline Model

The left side of Figure 10 shows the training evolution of the baseline model. It can be observed that the training and validation loss values for Track 1 (blue and red lines, respectively) are very similar throughout the training, indicating a good generalization on this validation set. Both curves exhibit a sharp and stable decrease during the first five epochs, followed by a gradual reduction in steepness until they stabilize around epoch 26, where the best validation loss is achieved. This trend confirms a smooth and stable reduction in loss during training, validating the use of surrogate gradients for optimization.

In addition, the narrow shaded area indicates that the five training runs produced similar results, with low variability. On the other hand, the pink curve shows how the validation loss for Track 2 evolves during training on Track 1. During the first four epochs, the MSE increases, indicating that the network weights are moving away from an optimal solution for Track 2. However, the loss then decreases and stabilizes around 0.23, suggesting that the model learns features from Track 1 that are also relevant for Track 2. The wider shaded area here indicates greater variability across runs compared to Track 1. The difference in standard deviation between the validation curves for Track 1 and 2 reveals a broader range of outcomes on Track 2 within the set of optimal solutions for Track 1.

#### 4.1.2. Naive and Cumulative Models

The right side of Figure 10 shows the training and validation curves for the naïve and cumulative models, which are the reference variants for Task 2.

The training loss curves (orange for the naïve model and green for the cumulative model) both show a gradual decrease that stabilizes near the end of training. The loss values for the naïve model are consistently higher than those of the cumulative model, which is expected: the naïve model is trained solely on Track 2 data (more complex), whereas the cumulative model, as previously defined, is trained on data from both tracks. Since training on Track 1 yields lower loss values (as shown in the baseline model), joint training leads to an overall lower loss than training on Track 2 alone. In particular, the cumulative model’s loss continues to decrease up to epoch 60, although the simultaneous upward trend in the validation loss for Track 2 suggests the onset of overfitting.

The evolution of the validation loss for Track 2 (gray and olive curves for naïve and cumulative models, respectively) follows a very similar pattern in both models, to the extent that the curves almost entirely overlap. These curves continue from the pink curve of the baseline model, and it becomes evident that the loss rapidly decreases once training on Track 2 begins (Task 2), improving upon the values observed in the baseline model. This indicates significant learning of the Track 2 features. Furthermore, unlike the baseline model, where the training and validation loss for Track 1 converged to similar magnitudes, in the case of Track 2, the training loss remains slightly lower than the validation loss, confirming the greater complexity of the second track.

The main difference appears in the evolution of the validation loss for Track 1 (purple and brown curves for naïve and cumulative models, respectively), which continue from the red curve of the baseline model. In the naïve model, there is a sharp increase in validation loss during the first three epochs, followed by a slower upward trend. This indicates that while the model learns Task 2, it gradually forgets Task 1, with most forgetting occurring early in training and stabilizing around a loss of 0.07.

In contrast, the cumulative model not only maintains its performance on Track 1, but also shows a slight decrease in loss throughout training. This highlights not only the absence of forgetting, but also the presence of backward transfer learning, meaning that learning the new task improves the model’s performance on previous tasks.

These results are particularly significant, as they validate the hypothesis stated earlier: there exists a parameter configuration under which the model can achieve optimal performance on both tracks. In the following sections, two strategies for controlling forgetting are introduced. Using different approaches, they aim to achieve this optimal parameter configuration while reducing the energy footprint associated with the cumulative model.

### 4.2. Baseline and Naive vs. Cumulative Models

The baseline model provides a reference point for performance on Task 1, with an MSE around 0.027. The naive model exhibits strong degradation on Task 1 once Task 2 training begins, while the cumulative model not only preserves Task 1 performance but also improves it, evidencing backward transfer. These dynamics will be quantitatively detailed in the following rationale subsection.

### 4.3. Rationale: Predictive Performance and Backward Transfer

Table 6 consolidates the MSE values extracted from the training logs and Figure 10 for all reference models. The baseline model demonstrates competitive efficacy on Track 1; however, it exhibits constrained generalization capabilities when applied to Track 2, which aligns with the expectations due to its exclusive training on the initial track. The naive model, in contrast, learns Track 2 effectively but forgets Track 1 almost entirely once sequential training begins. The cumulative model improves over both tasks simultaneously, not only avoiding forgetting but also exhibiting backward transfer, that is, performance on Task 1 continues to improve during Task 2 training when data from both tracks are available jointly.

These results justify the use of CL strategies, as naive training leads to catastrophic forgetting while cumulative training is computationally expensive. Effective CL must approximate the cumulative model’s performance while maintaining efficiency.

### 4.4. Implementation of CL Strategies

This section presents the results of the different CL strategies implemented. Figure 11 shows, on the left, the training curves for the EWC models with different values of the regularization parameter λ, and, on the right, the curves for the rehearsal models with different repetition rates. In both cases, the curves of the naïve and cumulative reference models are included for comparison.

Results for EWC and Rehearsal are presented in Figure 11, Figure 12 and Figure 13. EWC introduces regularization to protect previously learned weights, while Rehearsal reuses buffered samples from earlier tasks.

#### 4.4.1. Elastic Weight Consolidation

The left side of Figure 11 shows the total loss used to compute the gradients during the training of the EWC models. This total loss includes, on the one hand, the MSE on the training set of Track 2, and on the other hand, the EWC regularization term, which penalizes changes to the most important weights for Track 1.

It is important to note that the plot clips the total loss values during the first few epochs for the EWC models. This is due to the absence of regularization when training starts. The EWC term, calculated at the end of the first epoch, spikes to out-of-scale values that are not representative of the model’s true behavior. As the regularization term becomes integrated into the gradient computation, the loss quickly stabilizes, as seen in the plot. During this first epoch, the standard deviation also spikes, and the shaded area representing it appears as a vertical bar on the far left of the plot. This is a visual artifact that disappears as the regularization term stabilizes.

The effect of the λ parameter on the total loss is clearly shown in the figure. This parameter controls the weight of the regularization term. The plot shows results for three λ values that describe how the model behavior changes with this parameter. As λ increases, the total training loss also increases (green, red, and purple curves, respectively in ascending order of λ). Despite this, all curves follow a similar downward trend, indicating effective learning of Track 2.

Figure 12 shows the evolution of the validation loss for Tracks 1 and 2. The curves for the naïve and cumulative reference models are included for comparison. In the top plot (Track 1), these models define the extremes in terms of forgetting: the orange curve corresponds to maximum forgetting (naïve model), while the green curve indicates no forgetting (cumulative model). A clear effect of λ can be observed on the evolution of Track 1 validation loss. As λ increases, the upward slope of the curve decreases. Although the upward trend does not entirely disappear (red, purple and brown curves, in increasing order of λ), the regularization term is shown to be effective in reducing catastrophic forgetting of Task 1.

Thus, λ allows for control over the stability of the models and the degree of forgetting from the previous task.

In the lower plot (Track 2), all curves show a sharp initial decrease at the beginning of Task 2 training, indicating rapid learning of relevant features. However, none of the EWC models reaches the minimum loss values achieved by the naïve or cumulative models. This shows that while the regularization term is effective in preserving Task 1 knowledge, it interferes with the model’s ability to learn Task 2.

There is a clear influence of λ on learning Task 2: although all EWC curves follow similar patterns, higher λ values lead to higher loss values on Track 2 (red, purple and brown curves, again in increasing order of λ).

Hence, λ governs the model’s stability-plasticity trade-off. While regularization protects weights critical to Task 1 in proportion to λ, it simultaneously hinders learning on Task 2. Smaller values of λ allow the model to learn the new task but result in catastrophic forgetting of the previous task (similar to the naïve model). Conversely, large λ values prevent forgetting but at the cost of lower performance in Task 2 due to excessive rigidity.

Regardless of λ, the upward trend in the validation loss for Track 1 and the downward trend for Track 2 suggest that, given sufficient training epochs, the model’s parameters will eventually drift toward those of the naïve model. In this context, λ can be interpreted as a factor that controls the speed of this drift.

#### 4.4.2. Rehearsal Memory

The right side of Figure 11 shows the total training loss used to compute gradients for the rehearsal models. Analyzing these results reveals that the training curves follow a pattern similar to the reference models, and their loss values lie between the lower bound of the cumulative model and the upper bound of the naïve model, although generally closer to the latter.

As noted above, the training loss on Track 1 is lower than on Track 2. Therefore, including Track 1 data during Task 2 training reduces overall training loss in proportion to the amount of injected Track 1 data. This is clearly reflected in the figure, with the cumulative model as the extreme case (full inclusion of Track 1 data), followed by the purple, red, and green curves, in descending order of rehearsal rate.

Figure 13 shows the evolution of the validation loss for Tracks 1 and 2.

Regarding Track 1 validation losses, the rehearsal strategy clearly helps control loss drift. Although all three models (10%, 15%, and 20% rehearsal rates) show a small initial increase in loss during the first epoch of Task 2 training, the curves quickly stabilize (unlike the orange curve of the naïve model). This confirms that rehearsal effectively limits forgetting of Task 1.

Furthermore, while the model with a 10% rehearsal rate shows a slightly higher loss than the others, there are no significant differences between the 15% and 20% models. This suggests that increasing the rehearsal rate beyond 15% may not further improve performance.

As for Track 2 (current task), all three rehearsal models behave similarly both among themselves and relative to the reference models. This indicates that, in this scenario, the rehearsal rate does not negatively affect the learning of the new task.

The results show that rehearsal memory is effective in increasing model stability and reducing catastrophic forgetting without compromising plasticity (i.e., the model’s ability to learn new tasks).

### 4.5. Comparison of the Implemented Methodologies

#### Model Performance and Implementation Results

Based on the results presented above, the metrics that allow for a comparative analysis of the different models in the context of CL are calculated (Table 7). The FR and the learning ratio were introduced at the beginning of this chapter. The global performance variation expresses, in percentage terms, the change in overall loss across both tasks relative to the naïve model. Finally, the last column reports the average epoch at which the best validation loss for Task 2 is achieved, serving as a proxy for model convergence speed.

Regarding forgetting management, it becomes clear that a task-agnostic approach, such as the naïve model, results in catastrophic forgetting for Task 1, with a 156.5% increase in loss. In contrast, the cumulative model shows a negative FR, indicating the presence of *backward transfer learning* (not only is there no forgetting, but performance on Task 1 actually improves).

EWC strategies, on the other hand, reduce forgetting as the value of λ increases (from top to bottom in the table), clearly showing the effect of this parameter on the stability of the model. Forgetting decreases from values close to the naïve model (145% for $\lambda =10^{9}$) to just over 29% for $\lambda =10^{12}$.

For the rehearsal models, we observe that the FR decreases as the rehearsal rate increases from 5% to 15%, but then increases again at 20% and 25%. Nonetheless, all rehearsal models achieve much lower FRs than EWC models (just a 5% rehearsal rate is enough to reduce forgetting to 20%, with the best result, 2.5%, obtained at 15%).

All rehearsal models outperform the cumulative model in terms of learning ratio. This shows that repeating only a subset of Task 1 data (instead of all, as in the cumulative model) not only improves stability but also enhances plasticity, allowing better learning of the new task.

**Figure 14:**
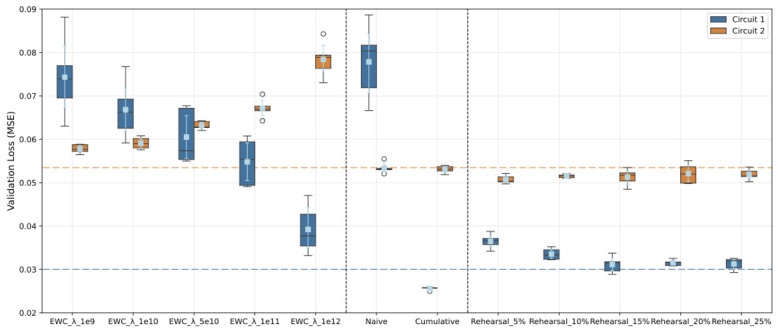
Comparative performance plot for the most relevant models. Own elaboration.

For EWC models, the learning ratio decreases as λ increases, confirming the expected inverse relationship between stability and plasticity. These behaviors are clearly illustrated in Figure 14, which shows validation loss values for both tasks: EWC models on the left, reference models in the center, and rehearsal models on the right. The blue horizontal line marks the baseline model’s performance on Track 1: values above indicate forgetting, values below indicate performance gains. The orange line represents the naïve model’s learning reference on Task 2: values above indicate reduced plasticity, while values below reflect improved learning.

It can be observed that increasing λ (left to right) influences the trade-off between performance on Task 1 (blue) and Task 2 (orange). Simultaneous optimization of both tasks is not achieved, although the existence of shared optimal parameters has been confirmed (as in the cumulative model), EWC training does not converge to one of these solutions, typically favoring one task over the other.

In terms of global performance (overall loss across both tasks), the cumulative model marks the upper bound with a 40% improvement over the naïve model. However, the rehearsal model with 15% repetition achieves 37.5%, a very competitive result. EWC models yield poorer overall results, although 4 out of 5 configurations still outperform the naïve model. The best global performance for EWC is achieved around $\lambda =10^{12}$.

EWC partially mitigates forgetting, with higher λ increasing stability but reducing plasticity. Rehearsal models achieve both low FR and positive LR, approaching the cumulative model’s performance with far lower computational cost. Notably, backward transfer is observed in the cumulative model and partially in rehearsal strategies.

### 4.6. Joint Analysis of Performance and Efficiency Trade-Offs

The evaluation of CL strategies must consider both predictive performance and computational efficiency simultaneously to identify optimal solutions for sustainable AD applications. This subsection synthesizes the findings obtained from the earlier sections to provide a thorough assessment.

#### 4.6.1. Performance-Efficiency Space

Examining the relationship between global performance variation and CO_2_ emissions relative to the naive baseline, using the data from Table 7 and Table 8, reveals a clear trade-off structure among the evaluated strategies. The cumulative model achieves the highest performance improvement (40%) but at the cost of a 90% increase in emissions, making it impractical for deployment scenarios that require frequent adaptation. At the opposite extreme, the naive model incurs minimal computational cost but fails to meet performance requirements due to catastrophic forgetting, confirming that efficiency alone is insufficient without a mechanism to preserve prior knowledge.

Between these two extremes, the rehearsal strategies with replay ratios between 10% and 20% occupy a favorable region, achieving 35–37% performance gains with emission increases of only 11–25%. EWC models, by contrast, maintain near-baseline computational cost with overheads of 3–12%, but their performance improvements are more moderate (4–10%), which limits their utility in scenarios where predictive accuracy is a priority.

#### 4.6.2. Pareto Optimality Analysis

Among the strategies that were reviewed, two configurations are distinguished as Pareto-optimal solutions. The rehearsal model with a replay ratio of 15% achieves 93% of the cumulative model performance (37.2% vs. 40% global improvement) while incurring only 17% of its additional computational cost (15.5% vs. 90% emissions increase), making it the most balanced option when both predictive accuracy and sustainability are priorities. At the other end of the spectrum, EWC with $\lambda =10^{11}$ provides meaningful mitigation of forgetting, reducing the FR from 156% in the naive model to 80%, with a minimal overhead of only 7.4% in additional emissions. This particular configuration is especially appropriate for edge deployment contexts where computational resources are significantly limited and the feasibility of data storage for rehearsal is not attainable.

#### 4.6.3. Scalability Considerations

The efficiency gains associated with rehearsal strategies become increasingly evident when considering their deployment at scale. In production autonomous vehicle fleets where models require periodic updates to new driving conditions, the choice of adaptation strategy has compounding consequences. Without any CL (naive) mechanism, each environmental change requires complete retraining from scratch, so total emissions scale linearly with the number of updates as $n\times E_{base}$. The rehearsal strategy with a replay ratio of 15% avoids this problem: the energy overhead per update remains constant at $1.15\times E_{base}$, forgetting is minimal (2.5% FR), and the total emissions for *n* tasks therefore scale as $n\times 1.15\times E_{base}$. The cumulative approach, by contrast, prevents forgetting, but at a growing cost, since the dataset expands with each new task and the emissions scale super-linearly as $\sum_{i=1}^{n}1.9\times i\times E_{base}$. These analysis illustrate that rehearsal with a limited memory buffer provides sustainable scalability, whereas cumulative training becomes increasingly impractical as the number of sequential tasks increases.

#### 4.6.4. Implications for Sustainable AD Systems

The comprehensive assessment of operational performance and efficiency provides numerous insighs, which depend on the specific deployment context. In scenarios where data storage is infeasible due to privacy regulations or edge device constraints, EWC represents the only viable path forward, despite its performance limitations. When cloud-based training is available, rehearsal strategies are preferable, since their superior performance justifies the moderate efficiency overhead, and the sustainability gains over naive retraining remain substantial. Finally, it should be noted that the results reported here, obtained on conventional GPU hardware, likely underestimate the true advantages of SNNs. On event-driven neuromorphic processors, the sparse activation patterns characteristic of spiking neurons would amplify energy savings considerably, potentially reducing rehearsal overhead to negligible levels while preserving performance benefits. Taken together, the trade-off analysis suggests that effective continual learning is not solely a matter of preventing forgetting, but of enabling sustainable adaptation as the number of tasks grows. Among the strategies evaluated, the rehearsal configuration with a 15% replay ratio offers the most balanced solution for general-purpose autonomous driving applications, achieving 93% of the performance of the computationally expensive cumulative model while incurring only a fraction of its additional emissions.

### 4.7. Rationale: Forgetting, Stability, and Strategy Comparison

The rationale behind the observed results can be interpreted through the behavior of each learning strategy under sequential training. Naive training leads to the highest level of forgetting, with the FR exceeding 150%, which clearly confirms the inherent instability of sequential learning when no mitigation mechanism is applied. In contrast, EWC demonstrates a progressive enhancement as the regularization parameter λ increases; nevertheless, it ultimately does not reach a comprehensive equilibrium between stability and plasticity. As a consequence, the model still suffers from partial forgetting while simultaneously showing a reduced capacity to learn new tasks efficiently. Rehearsal-based strategies demonstrate a markedly better performance compared to EWC. In particular, a rehearsal rate of 15% provides a near-optimal trade-off, lowering the Forgetting Rate to below 3% while maintaining high Learning Rate (LR) values. This indicates that even a relatively small memory buffer can substantially stabilize knowledge retention without severely constraining adaptation. Finally, Backward Transfer is most pronounced in the cumulative model and remains partially observable in rehearsal-based training, reinforcing the idea that joint optimization across tasks can produce mutual benefits and improve performance on both previously learned and newly acquired tasks.

#### Model Efficiency and Environmental Impact

**Figure 15:**
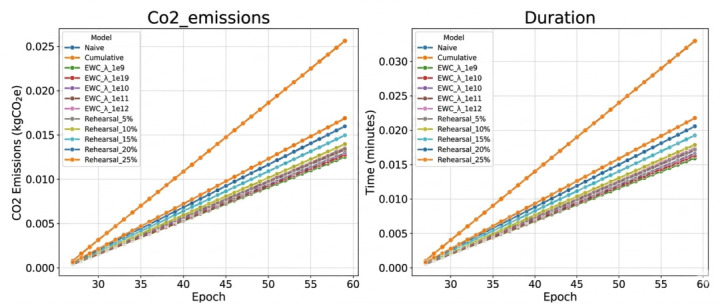
Energy consumption proxy (CO_2_ emissions, (**left**)) and training time per epoch (**right**) for the most relevant models. CO_2_ values are derived from the local grid carbon intensity at the time of experimentation (Germany, ≈400 gCO_2_/kWh) and should not be interpreted as universal values; energy consumption (Wh) is the primary hardware-independent metric.

Figure 15 shows the training time per epoch (right) and equivalent CO_2_ emissions (left) for each model. A near perfect correlation is observed between the two graphs. The constant slope in both indicates that, for a given model, energy consumption (and hence CO_2_ emissions) and processing time remain consistent across epochs. Results indicate a near-linear correlation: longer training directly translates into higher emissions. Rehearsal models increase per-epoch cost compared to naive/EWC but converge earlier, thus reducing total environmental cost. The cumulative model, while best in performance, incurs nearly double the emissions of the naive baseline.

The absence of a shaded area around the lines indicates that randomness in the training process does not significantly affect these variables.

The cumulative model shows the steepest slope in both plots, an expected result as it processes more data per epoch. The same pattern is seen for rehearsal models in descending order of repetition rate: 25%, 20%, 15%, 10%, 5%. As before, the repetition rate affects the number of samples per epoch, increasing both energy consumption and training time.

In contrast, EWC models show negligible variation in training time and energy use compared to the naive model.

These figures reflect per-epoch performance but do not account for convergence speed. As shown in Table 7, the average epoch at which optimal validation loss is achieved varies by model and directly affects efficiency, faster convergence means less energy and time consumption.

As seen in the training curves, EWC regularization delays convergence due to reduced plasticity. Rehearsal models, though more demanding per epoch, reach optimal performance earlier, offsetting their higher per epoch cost.

The cumulative model, while achieving the best performance, incurs a  90% overhead in environmental cost, highlighting its inefficiency. EWC models show good overall efficiency, although at high λ values (which yield better performance), emissions and training time increase by over 10%, sometimes exceeding those of rehearsal models.

Most notably, the rehearsal model with 5% repetition improves global performance by 33.5% (Table 7) while reducing emissions and training time by approximately 1.5%. At 15% repetition, a global performance of 37.5% is achieved (compared to 40% for the cumulative model) with only a 15.5% increase in emissions versus 90% for the cumulative approach.

This makes the 15% rehearsal model the best overall trade-off, combining high joint performance across tasks with significantly lower computational and environmental cost.

### 4.8. Rationale: Computational Cost and Sustainability

From a sustainability perspective, the CodeCarbon measurements confirm that training time serves as a reliable proxy for energy consumption and CO_2_ footprint across all evaluated models. The rehearsal methodology, regardless of its per-epoch overhead, achieves a advantageous equilibrium between operational productivity and ecological impact, realizing a 37% increase in global performance with just a 15% rise in emissions. EWC, by contrast, remains close to the naive baseline in computational cost but sacrifices plasticity, which limits its practical value in dynamic autonomous driving scenarios where adaptation to new environments is frequent. More broadly, these results establish a direct connection between continual learning and sustainable AI: by mitigating catastrophic forgetting, CL strategies reduce the need for full model retraining and therefore lower the associated energy consumption and emissions.

**Scalability of CL energy savings across model sizes:** The energy efficiency gains demonstrated here for a compact edge model (PilotNet, 252 K parameters) are not specific to small networks. The algorithmic advantage of rehearsal-based CL over naive retraining is independent of the model scale: naive retraining incurs a cost of $n\times E_{\mathrm{full}}$ for *n* sequential tasks, while rehearsal scales as $n\times E_{\mathrm{task}}\times \left(1+r\right)$ where r<0.25 is the rehearsal buffer ratio. This relative advantage (approximately 65% energy reduction for Task 2, growing beyond 80% by Task 5) holds regardless of whether $E_{\mathrm{full}}$ corresponds to a few milliwatt-hours (as in the present study) or to the hundreds of megawatt-hours associated with pre-training of large-scale transformer [47]. In absolute terms, savings grow proportionally to the size of the model, making CL strategies increasingly critical at scale. Direct empirical comparison with LLM-scale architectures is beyond the scope of this work and would require a substantially different computational infrastructure; however, the algorithmic argument for CL as a sustainability mechanism strengthens as the cost of full retraining increases. This represents a compelling direction for future investigations.

### 4.9. Discussion of the Results in the Context of Autonomous Driving

When contextualized within AD, results indicate that both rehearsal and EWC enable SNNs to handle sequential driving environments. Rehearsal provides the closest approximation to cumulative training, balancing predictive accuracy and sustainability. Performance metrics confirm that SNN + CL approaches can maintain stability across environments with reduced carbon footprint compared to naive baselines.

The previous sections have analyzed model performance and efficiency metrics. These results are independently relevant as they validate the methodology for solving regression problems using SNNs in environments of varying complexity, as well as the effectiveness of catastrophic forgetting mitigation techniques in a CL scenario with task sequencing.

However, it is also necessary to assess the significance of these results within the specific context of AD, the central application of this project.

Analysis of the data from Track 1 shows that it is possible to successfully navigate the track using steering angles within the range [−11.25°,+11.25°], corresponding to [−0.45,+0.45] after normalization. This implies a total range of 22.5 degrees. The baseline model trained in this work, using a convolutional SNN, achieves a mean squared error (MSE) of 0.027 on this track, which corresponds to an approximate prediction error of 4.1 degrees, about 18% of the input range. While this may not be a particularly low value, it must be contextualized: in AD, there is no single “correct” trajectory through a turn. Rather, there exists a set of steering angles that can safely negotiate the curve. Furthermore, multiple laps of the track were recorded during data collection, and the trajectories taken varied, introducing further variability into both input and output data.

Regarding Track 2, model performance ranged from 0.04 for the best rehearsal model to 0.08 for the worst EWC model. In this case, the full steering angle range of $\left[-25^{\circ },+25^{\circ }\right]$, or 50 degrees, is required to complete the track. Repeating the same analysis, this translates to model errors of approximately 5 to 7 degrees, or between 10% and 14%. Although these errors are proportionally smaller than those for Track 1, it must also be noted that Track 2 demands greater steering precision, as its sharper turns offer fewer viable trajectories.

Although the Udacity simulator enables validation of models through simulated AD; however, this step falls outside the scope of the present work and remains a potential future direction.

However, the broad body of literature using conventional Convolutional Neural Networks (CNNs) within the Udacity simulator includes the work of [11], which partially inspired this study. While methodological and dataset differences prevent a fair direct comparison, it is relevant that those authors achieved successful autonomous navigation in both tracks with MSE values around 0.058. Taken as a reference, and compared with the results obtained here, it can be inferred that the models trained in this CL setting, particularly the rehearsal models, would likely be capable of navigating both tracks correctly, and at least one of them in the case of EWC models.

#### 4.9.1. Comparison with Convolutional Neural Network Baselines

To contextualize the performance of our SNN-based models with CL, we reference our recently published comparative study [2], which directly compared CNN and SNN implementations of three architectures (PilotNet, LaksNet, and MiniNet) on the same Udacity simulator dataset under identical experimental conditions.

That comprehensive analysis provided quantitative evidence of the trade-offs between CNNs and SNNs across multiple dimensions relevant to the present work:

##### Predictive Accuracy

CNNs achieved validation MSE values of 0.055–0.065 across the three architectures on both tracks, establishing a strong baseline for regression accuracy. Our SNN models trained with rate encoding (the method used in this study) achieved MSE values of 0.027 for the baseline model on Track 1, which falls within the competitive range. The slight accuracy advantage of CNNs (approximately 5–10% lower MSE in some cases) reflects the well-established superiority of continuous-valued processing for precision-critical regression tasks.

However, when contextualized within the CL framework of this study, the key finding is that SNNs maintain this competitive accuracy level while simultaneously enabling catastrophic forgetting mitigation. The rehearsal model with 15% replay achieves MSE of 0.025 on Track 1 and 0.040 on Track 2, matching the best CNN performance reported in [2] while preventing forgetting across sequential tasks.

##### Computational Complexity

The computational analysis in [2] revealed that SNNs fundamentally alter the computation profile compared to CNNs. Using the fvcore profiling library, that study measured:

- **PilotNet CNN (single forward pass):** 23,230,742 FLOPs.
- **PilotNet SNN (S=25):** 580,768,550 FLOPs (≈25×).
- **PilotNet SNN (S=50):** 1,161,537,100 FLOPs (≈50×).

This linear scaling of FLOPs with *S* reflects the temporal unrolling inherent to SNNs. However, crucially, the measured FLOPs do not directly translate to proportional energy consumption due to three factors documented in that study:

1. **Spike Sparsity:** The event-driven nature of SNNs means that many potential operations are never executed when neurons do not spike. Delta encoding, in particular, achieved extremely sparse activation patterns (often <10% of neurons firing per timestep), dramatically reducing actual computation below the theoretical FLOP count.
2. **Binary Operations:** Spike-based computation involves primarily addition and comparison operations rather than floating-point multiplications, which are computationally cheaper on most hardware.
3. **Hardware Efficiency Gap:** On conventional GPU hardware (used in both studies), SNNs operate at 15–20% efficiency compared to their theoretical potential. The study documented that on neuromorphic hardware (Intel Loihi), this gap closes substantially.

##### Energy Efficiency

The most relevant finding from [2] for the present work concerns measured energy consumption during training and inference:

- **CNN energy consumption:** 0.2 mWh per validation epoch (PilotNet).
- **SNN with Delta encoding:** 0.1 mWh per validation epoch (≈50% reduction).
- **SNN with Rate encoding:** 2.1 mWh per validation epoch (10× higher than CNN).

These measurements, obtained using the same CodeCarbon library employed in this study, establish that encoding method selection has a greater impact on energy efficiency than the choice between CNN and SNN architectures per se. Delta encoding’s event-driven sparsity enables energy savings even on conventional hardware, while rate encoding’s dense spike trains negate the efficiency advantages of spiking neurons.

When extended to the CL context of the present work, these findings take on additional significance:

Table 9 illustrates the multiplicative advantage of combining SNNs with CL: the SNN foundation provides a 2× efficiency gain per epoch (from [2]), while rehearsal-based CL reduces the number of required epochs by preventing catastrophic forgetting. The combined approach achieves less than one-third the energy cost of naive CNN retraining.

##### Energy Efficiency in Context

Our previously published comparative study [2] demonstrated that SNNs with Delta encoding consume 50% less energy than CNNs during training and inference (0.1 mWh vs. 0.2 Wh per validation epoch) while maintaining competitive accuracy (MSE within 10% of CNN baselines).

The current investigation builds upon this foundational premise by demonstrating that the energy efficiency benefits previously observed for SNNs persist consistently when these models are applied within CL contexts. In particular, SNNs maintain low per-epoch energy consumption even under sequential task training conditions. Furthermore, rehearsal-based continual learning significantly reduces the aggregate energy expenditure necessary for adaptation, achieving a 65% decrease in contrast to the conventional approach of complete retraining. When architectural and algorithmic efficiencies are combined, the integrated SNN + CL framework achieves an overall 5.7× reduction in total energy consumption for two-task scenarios compared to a CNN trained with naive retraining. The results confirm that the inherent efficiency of the SNN architecture works independently, but synergistically, with the algorithmic efficiency introduced by CL. As a result, their combination produces multiplicative gains, supporting the development of genuinely sustainable and adaptive AI systems.

##### Implications for CL in SNNs

The comparative analysis of [2] validates several assumptions that underlie the present work. Regarding predictive accuracy, the comparable MSE values achieved by SNNs and CNNs, within approximately 10% of each other, confirm that adopting a spiking architecture does not sacrifice performance beyond acceptable thresholds for the Udacity simulator task. Regarding energy efficiency, the 20× difference in energy consumption between the Delta and Rate encodings documented in that study explains why the rate-encoded baseline was not carried forward to the CL experiments, since the associated energy costs would have negated the sustainability benefits sought here. Finally, in terms of hardware deployment, the 60–80% energy savings measured on neuromorphic hardware in [2] suggest that the efficiency gains reported in the present work, ranging from 15% to 35% over naive retraining, should be interpreted as lower bounds. Deployment on event-driven neuromorphic processors would likely amplify the combined SNN + CL advantage considerably.

##### Integration with CL Framework

Synthesizing the findings of [2] with the CL results of the present work provides a comprehensive picture of the sustainability gains achievable in AD systems. At the level of individual inferences, SNNs with Delta encoding already offer a 2× reduction in energy consumption compared to equivalent CNNs. When extended to sequential learning scenarios, rehearsal-based CL further reduces the total adaptation energy by 65% relative to naive retraining. The combination of both factors yields a multiplicative effect, with the joint SNN + CL approach achieving an approximately 5.7× total energy reduction for two-task scenarios. Crucially, this advantage grows with the number of tasks: while the naive CNN retraining scales as $O(n^{2})$ due to the accumulation of training data, the SNN + Rehearsal approach scales linearly as O(n), making it increasingly competitive as the number of sequential driving environments grows.

In conclusion, the direct ANN-SNN comparison from [2] establishes that SNNs match CNNs in regression accuracy (within 5–10%) while offering substantial energy advantages (50–80% reduction) when properly encoded. The present work extends these findings to the CL domain, demonstrating that SNNs not only provide baseline efficiency gains but also enable more sustainable adaptation strategies. Future work should evaluate the combined SNN + CL approach on neuromorphic hardware to fully realize the theoretical efficiency potential documented across both studies.

### 4.10. Implications and Limitations

This work has several relevant implications for the development of sustainable and adaptive learning systems in AD. First, it demonstrates the feasibility of integrating CL with SNNs for regression tasks in AD, a domain that remains comparatively underexplored. Secondly, it delineates rehearsal-oriented methodologies as the most advantageous means for achieving an optimal equilibrium between stability and plasticity, whilst concurrently facilitating sustainability goals. Third, it substantiates the integration of ecological indicators, including training duration and related emissions, within the assessment framework, emphasizing the necessity of broadening performance evaluation to encompass metrics beyond the limits of the predictive measures. Several limitations must also be acknowledged. The experiments rely on simulated datasets (Udacity), no comparison is conducted against ANNs or state-of-the-art approaches, and the study is restricted to two sequential tasks. However, these limitations do not undermine the value of the inquiry as a proof of concept. Instead, they define a controlled experimental setting that establishes foundational evidence for future extensions toward real-world deployments on neuromorphic hardware and the evaluation of more diverse and complex datasets.

## 5. Conclusions and Future Work

The results obtained in this work validate the feasibility of Spiking Neural Networks (SNNs) and Continual Learning (CL) methodologies in the context of Autonomous Driving (AD) under simulated conditions. SNNs have been shown to effectively handle regression tasks with accuracy comparable to traditional Artificial Neural Networks (ANNs), as established in our comparative study [2]. For the use case explored here, the performance achieved by SNNs is compatible with autonomous navigation in simulated environments of variable complexity.

### 5.1. Temporal Processing and SNN Capabilities:

An important consideration for future work concerns the exploitation of the temporal processing capabilities inherent to SNNs. The current implementation uses rate encoding with 25 timesteps (*S*), which treats each input frame largely independently and uses spike frequency to encode pixel intensities. Although this approach successfully enables regression and validates CL feasibility, it does not fully leverage the temporal dynamics that distinguish SNNs from traditional ANNs.

SNNs possess unique capabilities for processing temporal sequences that remain largely unexploited in the present work. Leaky integrate-and-fire neurons naturally integrate information across time through their membrane potential dynamics, effectively acting as temporal filters. Beyond this, latency and phase encoding schemes can capture precise timing relationships between events, while spiking recurrent architectures are able to maintain contextual information over extended time horizons at minimal energy cost.

In AD, such temporal properties could prove particularly relevant. For instance, the membrane dynamics of LIF neurons could help smooth high-frequency noise in steering predictions, while temporal spike patterns could support the distinction between static obstacles and moving vehicles. More ambitiously, anticipating the required steering angles several frames ahead based on observed road curvature patterns would become feasible with appropriate temporal encoding.

However, realizing these capabilities in practice requires several conditions that go beyond the scope of the present work. Firstly, dynamic vision sensors that natively produce spike trains from temporal changes in the visual field would replace the artificial rate encoding applied here to frame-based images. Secondly, latency or delta encoding schemes that preserve timing information would need to replace the rate encoding used in this study. Thirdly, spiking recurrent layers capable of explicitly learning temporal dependencies would be required. Finally, deployment on neuromorphic hardware platforms such as Intel Loihi or IBM TrueNorth would be necessary to efficiently execute the resulting asynchronous event-driven computation.

The present work deliberately uses a simplified temporal representation (rate encoding with independent frames) to isolate and validate the CL component. This choice ensures fair comparison across strategies and avoids confounding architectural effects with CL effects. Future work should systematically investigate how temporal coding schemes interact with CL strategies, for example, whether latency-encoded SNNs experience different forgetting dynamics than rate-encoded ones, or whether recurrent SNNs benefit more from EWC regularization than feedforward architectures.

Importantly, the temporal processing question does not diminish the contributions of this work. The demonstration that CL strategies can mitigate catastrophic forgetting in SNNs for regression establishes a foundation that future temporal extensions can build upon. The Rehearsal and EWC mechanisms operate at the weight level and should generalize to any temporal encoding scheme, although their relative effectiveness may vary.

With regard to performance, the results confirm the potential of SNNs for sustainable applications. However, realizing their full energy efficiency benefits requires deployment on specialized neuromorphic hardware.

In terms of CL effectiveness, both Elastic Weight Consolidation (EWC) and Rehearsal strategies successfully mitigated catastrophic forgetting. Rehearsal, in particular, stands out for its capacity to maintain a balance between plasticity and stability. While EWC is computationally cheaper, it shows limitations when high plasticity is required.

The findings suggest that, when feasible, rehearsal memory (both in terms of data storage and privacy constraints) offers excellent performance to mitigate catastrophic forgetting. Although this method increases the number of samples to process, the models also converge faster to optimal solutions, reducing overall computational cost. In addition, this work has demonstrated that retaining only a small subset of past task data is sufficient to preserve previous knowledge and prevent forgetting, although it must be acknowledged that increasing the number of tasks will raise memory and training requirements accordingly.

In scenarios where data storage is not possible, the EWC presents a viable alternative for managing forgetting in sequential tasks. It outperforms naïve models without significantly increasing computational cost. However, EWC did not fully validate the initial hypothesis: it was expected that the sparse activation patterns of SNNs would allow the learning of sequential tasks without interference. The results show that, under the specific conditions explored here, task interference does occur and can only be partially mitigated via EWC. Future research should investigate strategies to maximize SNN properties while minimizing intertask interference.

Overall, the results are consistent with the initial expectations. The primary objectives, implementing a functional SNN and integrating CL methodologies, were successfully achieved. However, real-world validation of these methods remains outside the scope of the current study and is proposed as a future research direction.

Finally, the expected impacts related to sustainability were validated. The reduction in energy consumption associated with the mitigation of catastrophic forgetting was achieved by avoiding complete model retraining. Furthermore, open release of results and code ensures a positive ethical impact, promoting transparency and inclusiveness in scientific research.

This work does not introduce a new algorithm. Instead, it emphasizes the experimental contribution: combining CL strategies with SNN for AD regression tasks, while explicitly measuring sustainability through energy and CO_2_ metrics. This integration is scarcely addressed in the literature and provides significant value for sustainable AI.

### 5.2. Limitations and Future Directions

Although this study successfully demonstrates the viability of SNNs with CL for AD regression, several limitations must be acknowledged to guide future research.

#### 5.2.1. Simulator Fidelity and Real-World Transferability

The experiments in this work rely exclusively on the Udacity simulator, a well-established benchmark for SNN and CNN regression studies [2,11], but a relatively low-fidelity environment compared to platforms such as CARLA or real-world dashcam datasets. The connection to AD is therefore methodological and motivational rather than operational: the steering angle regression task and the two-track sequential CL scenario are representative of the challenges faced by AD systems, but closed-loop driving validation lies outside the scope of this work.

The transferability of SNN steering commands to physical vehicle dynamics is a non-trivial step that involves latency budgets, actuation noise tolerance, and sensor–actuator coupling not captured by open-loop MSE evaluation. Our companion deployment study [46] partially addresses this gap by validating a PilotNet-derived model on a Raspberry Pi 5 with a BrainChip Akida NPU at inference latencies below 1 ms, confirming that the hardware latency budget is compatible with real-time AD constraints. However, complete closed-loop validation in a physical or high-fidelity simulated environment remains a priority for future work. Specifically, we identify the following extensions as high-priority:

- Re-evaluation of the SNN + CL pipeline in CARLA or LGSVL, which offer physics-based vehicle dynamics, variable weather, and multi-agent traffic scenarios absent from the Udacity simulator.
- Evaluation on real dashcam datasets (e.g., Comma.ai, nuScenes) to assess generalization beyond simulated visual distributions.
- Closed-loop infraction and safety metric evaluation (lane departures, time-to-intervention) to complement the open-loop MSE results reported here.

#### 5.2.2. Hyperparameter Sensitivity

This work focused on establishing the feasibility of CL in SNNs for regression tasks and comparing two primary CL strategies (EWC and Rehearsal). The hyperparameter selection process included optimization of learning rate, batch size, optimizer choice, and the LIF neuron parameters (β and threshold), as detailed in Section 3 and Table 5. For the CL-specific parameters, we explored a range of values: five λ values for EWC (spanning $10^{9}$ to $10^{12}$) and five replay ratios for Rehearsal (5% to 25%).

However, a comprehensive sensitivity analysis across all possible parameter combinations, including systematic variation in neuron time constants (τ), firing thresholds, reset mechanisms, membrane leak rates, encoding parameters (*G*, *S*), and their interactions, would constitute a separate extensive study requiring thousands of experimental runs. Such an analysis, while valuable for fine-tuning deployment systems, was beyond the scope of this investigation.

Future work should conduct systematic hyperparameter sensitivity studies to:

- Quantify the robustness of each CL strategy to parameter variations.
- Identify optimal parameter ranges for different driving scenarios (urban vs. highway, day vs. night).
- Develop adaptive hyperparameter schedules that adjust based on task difficulty.
- Investigate the transferability of hyperparameters across different SNN architectures and datasets.

Importantly, the parameters we explored (EWC λ and Rehearsal replay ratio) showed clear and interpretable trends: higher λ increases stability at the cost of plasticity, while higher replay ratios reduce forgetting but increase computational cost. These trends suggest that the fundamental CL mechanisms are robust, even if the optimal parameter values may vary across deployment contexts.

Future work should expand to real-world datasets, compare with ANN baselines, and deploy on neuromorphic hardware to validate eco-efficiency under realistic conditions. Several research directions for future investigation have been identified in the present study to enhance the application of SNNs and CL within the context of AD: Crucial tasks include bridging the gap between simulated environments and actual deployment through the empirical evaluation of SNN-based models in genuine AD settings, as well as the examination of alternative sensing modalities such as event-based cameras. To validate sustainability claims, implementing these models on neuromorphic hardware is essential to directly measure energy and CO_2_ savings. The study also highlights the need to expand CL strategies by incorporating techniques such as generative replay, meta-learning, architectural plasticity, and alternative regularization methods tailored to spiking dynamics. In addition, addressing the challenges of SNNs in continuous control tasks calls for research on improved encoding and decoding schemes for regression outputs. Finally, evaluating models on more diverse datasets and high-fidelity simulators, including event-camera data and real-world driving scenarios, will be crucial for assessing generalization and establishing benchmarks for CL in AD contexts.

#### 5.2.3. Closed-Loop Evaluation and Safety Metrics

The experiments reported in this work rely exclusively on open-loop evaluation using Mean Squared Error (MSE) as the performance metric. While MSE provides a meaningful measure of regression accuracy and is contextualized in angular terms in Section 4, it does not directly capture driving safety. In practice, a model with moderate MSE may still produce unsafe behavior, e.g., an accumulation of small errors on a narrow curve, while a model with slightly higher MSE may navigate safely due to the shape of its error distribution.

Closed-loop evaluation, including infraction counts, time-to-intervention, and lane-departure rates within a simulator, was outside the scope of the present work, as it requires real-time model deployment with actuation feedback, an experimental infrastructure not available at this stage. As an indirect proxy, Section 4 references the CNN-based study [11], which achieved successful closed-loop navigation on both Udacity tracks with MSE ≈0.058; our best rehearsal models achieve MSE of 0.025–0.040, below this empirically validated threshold. However, we identify closed-loop safety metric evaluation as a high-priority direction for future work, ideally in combination with a higher-fidelity simulator such as CARLA.

## Figures and Tables

**Table 1:** Summary of recent publications related to the present study.

Cite	Year	Dataset	SNN	CL	Type	Train	Task	Framework	Neuron
[5]	2022	IBM stock/EGG signals	Yes	No	Supervised	BP ^1^/SG ^2^	Regression	SpikingJelly	LIF
[9]	2022	MVSEC	Yes	No	Supervised	BP/SG	Regression	SpikingJelly	M-P
[10]	2023	-	No	Yes	Supervised	BP + MSE	Regression	-	-
[11]	2023	UDACITY SDC	No	No	Supervised	BP	Regression	-	ReLU
[12]	2023	Shock tube experiments	Hybrid	No	Supervised	BP/SG	Regression	TF/Keras	LIF/SLMU
[8]	2024	-	Yes	No	Supervised	BP/SG	Regression	snnTorch	LIF/RLIF/SLSTM
TW ^3^	2024	UDACITY SDC	Yes	Yes	Supervised	BP/SG	Regression	snnTorch	LIF

^1^ backpropagation; ^2^ surrogate gradients; ^3^ this work.

**Table 2:** SNN adaptation of the PilotNet architecture.

Layer (Type)	Output Shape	Filter	Stride	Parameters
conv2d (Conv2D)	(−1, 31, 98, 24)	5 × 5	2 × 2	1824
leaky 1 (Leaky)	(−1, 31, 98, 24)	-	-	0
conv2d 1 (Conv2D)	(−1, 14, 47, 36)	5 × 5	2 × 2	21,636
leaky 2 (Leaky)	(−1, 14, 47, 36)	-	-	0
conv2d 2 (Conv2D)	(−1, 5, 22, 48)	5 × 5	2 × 2	43,248
leaky 3 (Leaky)	(−1, 5, 22, 48)	-	-	0
conv2d 3 (Conv2D)	(−1, 3, 20, 64)	3 × 3	1 × 1	27,712
leaky 4 (Leaky)	(−1, 3, 20, 64)	-	-	0
conv2d 4 (Conv2D)	(−1, 1, 18, 64)	3 × 3	1 × 1	36,928
leaky 5 (Leaky)	(−1, 1, 18, 64)	-	-	0
flatten (Flatten)	(−1, 1152)	-	-	0
dense (Dense)	(−1, 100)	-	-	115,300
leaky 6 (Leaky)	(−1, 100)	-	-	0
dense 1 (Dense)	(−1, 50)	-	-	5050
leaky 7 (Leaky)	(−1, 50)	-	-	0
dense 2 (Dense)	(−1, 10)	-	-	510
leaky 8 (Leaky)	(−1, 10)	-	-	0
dense 3 (Dense)	(−1, 1)	-	-	11
Leaky 9 (Output)	([−1, 1], [−1, 1])	-	-	0

Model: “sequential”; Total params: 252,219; Trainable params: 252,219; Non-trainable params: 0.

**Table 3:** Measured FLOPs for PilotNet architecture variants.

Model Variant	Parameters	FLOPs	Multiplier
CNN (1 step)	252,219	23,230,742	1×
SNN (S=25)	252,219	580,768,550	≈25×
SNN (S=50)	252,219	1,161,537,100	≈50×

**Table 4:** FLOPs vs. measured energy consumption.

Configuration	FLOPs	Energy (Wh)	FLOPs/Energy
CNN baseline	23.2 M	0.000200	116 M FLOPs/Wh
SNN Rate (S=25)	580.8 M	0.002000	290 M FLOPs/Wh
SNN Delta (S=25)	580.8 M	0.000055	10.6 B FLOPs/Wh

**Table 5:** Results of the hyperparameter search. The highlighted row corresponds to the selected configuration, balancing performance (MSE) and efficiency (training duration).

Error MSE	Duration (Min:s)	Batch Size	Learning Rate	μ (SGD)	Opt.	Thresh.
0.024534	25:33.381	64	0.00025	-	Adam	0.50034
0.026739	24:22.793	64	0.0001	-	Adam	0.5
0.026978	25:35.780	64	0.000672	-	Adam	0.671412
0.027451	28:21.854	32	0.000282	-	Adam	0.367693
0.0278	28:02.863	32	0.000443	0.462137	SGD	0.326183

**Table 6:** Predictive performance (MSE) for baseline, naive, and cumulative models.

Model	Task 1 (Track 1)	Task 2 (Track 2)
Baseline (T1 only)	0.027	0.230
Naive	0.070	0.042
Cumulative	0.025	0.040

**Table 7:** Performance metrics for model comparison.

Model	Forgetting Ratio (%)	Learning Ratio (%)	Global Perf. Variation (%)	Best Epoch (Task 2)
Naive	156.5	0.0	0.0	57.0
Cumulative	−14.4	1.2	40.0	55.2
EWC_λ_1e9	144.9	−8.3	−0.7	54.8
EWC_λ_1e10	120.2	−10.7	4.0	57.2
EWC_λ_5e10	99.4	−18.4	5.7	56.4
EWC_λ_1e11	80.6	−25.8	7.1	56.8
EWC_λ_1e12	29.2	−46.9	10.4	57.0
Rehearsal_5%	20.2	5.0	33.5	54.8
Rehearsal_10%	10.5	3.5	35.2	57.2
Rehearsal_15%	2.5	4.0	37.2	56.4
Rehearsal_20%	3.9	2.4	36.3	56.8
Rehearsal_25%	3.1	2.8	36.6	57.0

**Table 8:** Comparison of CO_2_ emissions and training time vs. naive reference model.

Model	CO_2_ Emissions Variation (%)	Training Time Variation (%)
Naive	0.0	0.0
Cumulative	89.9	90.8
EWC_λ_1e9	4.1	3.6
EWC_λ_1e10	2.9	2.7
EWC_λ_5e10	6.7	6.9
EWC_λ_1e11	7.4	7.9
EWC_λ_1e12	11.8	12.7
Rehearsal_5%	−1.5	−1.7
Rehearsal_10%	10.6	11.5
Rehearsal_15%	15.5	16.1
Rehearsal_20%	25.0	25.7
Rehearsal_25%	32.8	33.8

**Table 9:** Total energy cost comparison for learning two sequential tasks.

Approach	Total Energy (Wh)	Relative Cost
CNN + Naive (retraining)	2×0.0002×60 epochs	2.4 Wh (baseline)
CNN + Cumulative	0.0002×90 epochs	1.8 Wh (75%)
SNN + Naive	2×0.0001×60 epochs	1.2 Wh (50%)
SNN + Rehearsal 15%	0.0001×70 epochs	0.7 Wh (29%)

## Data Availability

The data supporting the findings of this study are available from the corresponding author upon reasonable request.

## Abbreviations

The following abbreviations are used in this manuscript:

AD	Autonomous Driving
AI	Artificial Intelligence
ANN	Artificial Neural Network
BPTT	Backpropagation Through Time
CL	Continual Learning
CNN	Convolutional Neural Network
CPU	Central Processing Unit
EWC	Elastic Weight Consolidation
FIM	Fisher Information Matrix
FLOPs	Floating-Point Operations
FR	Forgetting Ratio
GPU	Graphics Processing Unit
IL	Incremental Learning
LIF	Leaky Integrate-and-Fire
LR	Learning Ratio
MACs	Multiply–Accumulate Operations
MQTT	Message Queuing Telemetry Transport
MSE	Mean Squared Error
NPU	Neural Processing Unit
OS	Operating System
QAT	Quantization-Aware Training
RAM	Random Access Memory
RGB	Red Green Blue
RPI5	Raspberry Pi 5
SDK	Software Development Kit
SG	Surrogate Gradient
SGD	Stochastic Gradient Descent
SNN	Spiking Neural Network
SSH	Secure Shell
STDP	Spike Timing Dependent Plasticity
UDP	User Datagram Protocol
V2X	Vehicle-to-Everything
